# Longitudinal Monitoring of Biomechanical and Psychological State in Collegiate Female Basketball Athletes Using Principal Component Analysis

**DOI:** 10.1155/2024/7858835

**Published:** 2024-04-03

**Authors:** Joshua A. J. Keogh, Matthew C. Ruder, Kaylee White, Momchil G. Gavrilov, Stuart M. Phillips, Jennifer J. Heisz, Matthew J. Jordan, Dylan Kobsar

**Affiliations:** ^1^Department of Kinesiology, Faculty of Science, McMaster University, Hamilton, ON L8S 4L8, Canada; ^2^Faculty of Kinesiology, Sport Medicine Centre, University of Calgary, Calgary, AB T2N 1N4, Canada

## Abstract

**Background:**

The growth in participation in collegiate athletics has been accompanied by increased sport-related injuries. The complex and multifactorial nature of sports injuries highlights the importance of monitoring athletes prospectively using a novel and integrated biopsychosocial approach, as opposed to contemporary practices that silo these facets of health.

**Methods:**

Data collected over two competitive basketball seasons were used in a principal component analysis (PCA) model with the following objectives: (i) investigate whether biomechanical PCs (i.e., on-court and countermovement jump (CMJ) metrics) were correlated with psychological state across a season and (ii) explore whether subject-specific significant fluctuations could be detected using minimum detectable change statistics. Weekly CMJ (force plates) and on-court data (inertial measurement units), as well as psychological state (questionnaire) data, were collected on the female collegiate basketball team for two seasons.

**Results:**

While some relationships (*n* = 2) were identified between biomechanical PCs and psychological state metrics, the magnitude of these associations was weak (*r* = |0.18-0.19|, *p* < 0.05), and no other overarching associations were identified at the group level. However, post-hoc case study analysis showed subject-specific relationships that highlight the potential utility of red-flagging meaningful fluctuations from normative biomechanical and psychological patterns.

**Conclusion:**

Overall, this work demonstrates the potential of advanced analytical modeling to characterize components of and detect statistically and clinically relevant fluctuations in student-athlete performance, health, and well-being and the need for more tailored and athlete-centered monitoring practices.

## 1. Introduction

Collegiate-level athletics have demonstrated unprecedented growth in participation over the past two-to-three decades [1–3]. Unfortunately, this increase in participation has been noted to be accompanied by an increase in injury burden [4, 5], which has been reported to be more pronounced in female athletes [6–8], and ultimately reduces the ability of an individual and a team to perform optimally during competition. Many of these sports-induced injuries are noncontact [1, 6, 7] and may be related to the exceedance of musculoskeletal (MSK) structure load tolerance from repeated bouts of vertical jumping, cutting, and pivoting tasks experienced during training and competition [1, 9–11]. Additionally, it has been demonstrated that between-limb differences (e.g., biomechanical deficiencies in strength, etc.) can continue throughout a seemingly successful rehabilitation and persist well after athletes have returned-to-sport [5, 12–15], and more limited evidence suggests that asymmetry may also be related to sports performance [16–23] and injury risk [18, 24–27] in healthy, competitive athletic populations.

MSK overuse injuries may be preventable by prospectively monitoring athlete workload, training intensity, and biomechanical deficiencies (e.g., jump asymmetry) [28–31]. However, biomechanics are often assessed in laboratory settings using proxies for sport-specific loading parameters using movements such as vertical jump testing [32–34], which might not necessarily be indicative of the biomechanical patterns that individuals exhibit in their sporting environments due to the principle of specificity [35–38]. Additionally, these traditional laboratory biomechanical assessments are costly and time consuming, which limits the practicality of employing these methodologies in regular, longitudinal athletic monitoring practices. To address this challenge, biomechanics has seen tremendous growth in the availability of accessible and more cost-effective modalities (e.g., inertial measurement units (IMUs) and portable force plate systems) that can be seamlessly applied in sport-specific settings to monitor individualized biomechanical patterns and fluctuations in real-world settings [39–42].

Nevertheless, assessing sports performance and injury susceptibility through a purely biomechanical lens may neglect important aspects of an athlete's adaptive potential [43]. For example, psychological stressors may be modifiable risk factors for injury and reinjury in and of themselves [44–51] or may add to the demands of a task in conjunction with the biomechanical stressors experienced [44, 52]. Remarkably, very limited research has directly examined the link between biomechanical outcomes and psychological state as it relates to sports performance and risk of injury, with even fewer research studies doing so longitudinally throughout several competitive seasons [53].

The complex and multifactorial nature of sports injuries [1, 7, 54–56] highlights the importance of monitoring athletes prospectively using a novel and integrated biopsychosocial approach. Specifically, defining a more comprehensive biomechanical profile consisting of on- and off-court patterns and contextualizing biomechanical changes with concurrent changes in psychological state might improve our understanding of specific underlying domains or facets that lead to sport injuries. However, in taking this approach, an issue may arise, namely, how best to summarize and interpret the multiple and diverse forms of data being collected [43, 57, 58]. Typically, key performance indicators for athlete monitoring are identified via evidence-based research or expert opinion [28, 29, 31, 34, 44]. However, this approach may overlook the presence of potentially significant variables that could contribute to the development of sports injuries [57, 59]. Alternatively, methods like the principal component analysis (PCA) are becoming more common for efficiently summarizing vast amounts of important information from numerous variables into a reduced number of key metrics or principal components (PCs) [57–62]. While this is a growing area of study, the application of the PCA in athlete monitoring programs to condense biomechanical profiles, including on- and off-court patterns, alongside concurrent changes in psychological state, remains unexplored.

The overarching aim of this project was to examine the utility of integrating on- and off-court biomechanical data into more holistic biomechanical metrics (e.g., PCs), with concurrent longitudinal monitoring of psychological state to contextualize these subject-specific biomechanical fluctuations. Specifically, data collected over two competitive collegiate female basketball seasons were used in a PCA to support the following research questions: (i) are PC scores derived from a biomechanical model (i.e., on-court and vertical jump data) significantly correlated with introspective, psychological state (e.g., self-reported pain, etc.) across a season and (ii) can we detect subject-specific meaningful changes in these measures using PCs and associated minimum detectable change (MDC) statistics?

## 2. Methods

### 2.1. Study Design

A longitudinal cohort design was used to collect repeated data on athletes across multiple seasons. Specifically, biomechanical and psychological data were collected in two consecutive seven-month collegiate female basketball seasons (i.e., the 2021-2022 and 2022-2023 competitive seasons) at McMaster University. These data were collected during three consecutive training phases, namely, one month of offseason training, two months of preseason training, and during the four-month regular season. Data were collected weekly, including CMJ testing and psychological state questionnaires administered on Monday mornings, while on-court biomechanical assessments with IMUs were completed during three weekly basketball practices. All data (i.e., CMJ, on-court IMU, and psychological state) were summarized as weekly averages. The correlation between the array of metrics, both on- and off-court, gave credence to the use of a PCA as it suggested that these metrics may have some redundancy and share commonalities to similar, underlying components of biomechanical movement patterns (Supplementary Table 1). Additionally, the reliability of the biomechanical PCs and introspective and psychological state metrics were assessed across five consecutive and unperturbed (i.e., no scheduled periods of intensified competition or extended rest that would implicate the consistency of these measures) weeks in the 2022-2023 preseason using the same statistical approach previously outlined in a similar work by our laboratory [63]. The results of the current reliability analysis can be found in Supplementary Table 2. After determining the need for and reliability of the biomechanical PCA, we applied this model to research questions (i) and (ii). This PCA model was developed from 2021 to 2022 data and applied to 2022-2023 season data to assess the relationship between biomechanical PCs and introspective and psychological state data and to highlight the ability to identify meaningful changes (i.e., red-flag) subject-specific alterations in these outcome measures.

### 2.2. Sample

Sixteen female collegiate basketball athletes (eight guards, five forwards, and three centers) from McMaster University who were free from MSK injury or disorder at initial screening volunteered to participate in the study: age 20 (2) years, height 178 (9) cm, body mass 73 (11) kg, and training experience 3 (1) years. Participants were informed of the potential risks, benefits, study protocol, and were made fully aware of their ability to withdraw from the study at any time. Written consent was obtained from all participants. This study was reviewed and approved by the university research ethics board.

### 2.3. Protocol

#### 2.3.1. Data Collected

First, four on-court impact acceleration asymmetry metrics (i.e., low- (1–5 g), moderate- (6−20 g), high- (21–200 + g), and total impact acceleration asymmetry [38]), impact load, step count, and average intensity (i.e., *n* = 7 on-court metrics) were assessed as weekly average values using peak resultant linear accelerations recorded with IMUs (iMeasureU, Vicon) placed bilaterally anterosuperior to the medial malleoli during on-court practices. Interlimb asymmetry metrics were chosen due to the previously identified implications to sport performance and risk of injury [16, 17], while impact load, step count, and average intensity were included given that the most frequent mechanism of injury in basketball relates to improper landings from vertical jumping or during change-of-direction-related tasks [1, 64], and these metrics may provide a surrogate measure for the volume of such on-court activities. Second, four between-limb CMJ asymmetry measures (peak braking-, propulsive-, and landing-force, and average braking rate of force development asymmetry [63]), peak power production during the braking and propulsive phases of movement, countermovement depth (CMD), time to takeoff, jump height (JH), and the modified reactive strength index (RSI mod = JH/time to takeoff) were assessed weekly with the CMJ without an arm-swing using a portable bilateral force plate system (Hawkin Dynamics) with a sampling frequency of 1000 Hz. This method of biomechanical assessment during vertical jump testing has been deemed valid when compared to the in-laboratory gold standard [65, 66]. Moreover, the reliability and ecological validity of these CMJ metrics were assessed in work paralleled to this investigation by our laboratory and were found to be highly reliable (ICC ≥ 0.90) when assessed longitudinally, while the asymmetry metrics were relatively independent of one another which highlighted the need to concurrently collect traditional and sport-specific asymmetry measures [38, 63]. Finally, weekly questionnaires were completed via Google Forms on the athletes own volition on Monday mornings using a questionnaire built by a multidisciplinary team of researchers in collaboration with the McMaster University Strength and Conditioning team. Specifically, this weekly questionnaire was composed of the following self-report measures: self-reported pain, sleep quality and sleep quantity, a general feeling scale, and academic workload, which were assessed using the Visual Analogue Pain Scale [67], a subsection of the Pittsburgh Sleep Quality Index [68], and a 0–10 Likert scale for feeling and academic workload, respectively. Self-reported pain was scored from 0 to 10, with 0 representing no pain, 2 representing mild pain, 4 representing moderate pain, 6 representing severe pain, 8 representing very severe pain, and 10 representing the worst pain imaginable. Athletes scored their perceived pain according to the body region (i.e., ankle, knee, hip, back, shoulder, quadriceps, hamstrings, glutes, calves, none, or other), while the pain measures that were utilized for statistical purposes were generalized to the lower extremity. Sleep quality and sleep quantity were separately scored from 0 to 3, with 0 representing very good and >7 hours, 1 representing fairly good and 6-7 hours, 2 representing fairly bad and 5-6 hours, and 3 representing very bad sleep quality and <5 hours of sleep for sleep quality and quantity, respectively. General feeling was scored from 0 to 10, with 0 representing very bad, 2 representing bad, 4 representing fairly bad, 5 representing neutral, 6 representing fairly good, 8 representing good, and 10 representing very good general feeling as a proxy for overall mental health. Last, academic workload was scored from 0 to 10, with 0 representing none, 3 representing light, 5 representing average, 7 representing heavy, and 10 representing an overwhelming academic workload.

#### 2.3.2. Testing Procedures

The CMJ testing and on-court session procedures have been previously outlined and can be found in other works conducted by Keogh and colleagues [38, 63]. Briefly, CMJ testing was conducted on Monday mornings after the completion of a low-to-moderate-intensity dynamic warm-up to prepare the neuromuscular system. Participants performed three CMJ attempts without an arm-swing, self-selected their CMD, were cued to jump as high and quickly as possible, and were provided a minimum of 30-seconds of rest between successive attempts [63]. On-court sessions were completed between two and three times per week in which IMUs were positioned bilaterally anterosuperior to the medial malleoli throughout 90- to 120-minute basketball practices [38]. CMJ outcome measures were calculated from the vertical ground reaction force data, and on-court outcome measures were obtained from the resultant accelerometer data from IMUs. All outcome measures were obtained from the respective manufacturer-provided software.

#### 2.3.3. Model Development

A total of seven months of data were collected during the 2021-2022 season, resulting in a total of 448 possible weekly observations (28 weeks × 16 athletes) of the abovementioned 22 variables. The resulting data matrix (e.g., 448 rows of observations by 22 columns of variables) was used to depict the variability in this dataset through the PCA accomplished using the “pca” function in MATLAB R2021a (MathWorks, Inc., Natick, MA, USA). This function utilizes the singular value decomposition approach which aims to consolidate commonalities or correlations between the original biomechanical variables by uniquely loading (i.e., rotating) them onto new variables called PCs. Given the varying scales of our biomechanical variables, standardization of variables (i.e., mean of zero and standard deviation of one) was required for the PCA [57, 69]. These newly developed PCs are uncorrelated with each other but can effectively represent overarching structures (i.e., principal components) within the data and the variability present. The PCs were derived based on the maximum variance explained in the data and presented in descending order from PC1 to n-PC (i.e., the number of PCs required to explain ≥90% of the data variance) [57, 69]. This method aligned well with other approaches to retain PCs (e.g., scree plot and 0.7 eigenvalue cutoff) [57, 69, 70]. In other words, the first PC highlights commonalities in variables that represent data with the greatest variability across the sample. Further, these new independent variables (PCs) depict scores for each individual relating to a more holistic or summarized assessment of important biomechanical data and, using the coefficients which define the interrelationship between original variables, can be computed weekly for each athlete during the 2022-2023 season to address research questions (i) and (ii).

### 2.4. Statistical Analysis

- *Association between biomechanical and introspective, psychological state*: The relationships of biomechanical PC scores to introspective, psychological state metrics were assessed using a repeated measures correlation statistical approach [71]. The repeated measures correlation was used to determine the common within-individual associations between these continuous variables (i.e., biomechanical PCs and psychological state), while ensuring that the assumption of independence of observations was not violated by controlling for the effect of between-individual variance [71].
- *Detection of Subject-Specific Fluctuations from Normative, Baseline Patterns*: standardized error of the measurement (SEM) and MDC statistics [72] were computed for red-flagging and identifying subject-specific statistically important levels of change in our biomechanical PCs and introspective, psychological state metrics across the 2022-2023 season. These MDC statistics were defined using preseason data as normative baseline values and were carried forth across the season to discern when subjects had exhibited fluctuations outside of their normative patterns demonstrated in the preseason. In turn, a red-flag event would occur if an athlete's data changed greater than the defined MDC, highlighting a deviation that is larger than the expected level of between collection variance for a given PC. This methodological approach has been proven reliable and was applied in a similar longitudinal use-case example by our laboratory. [63] All statistical analyses (*α* = 0.05, and *β* = 0.20) aside from the repeated measures correlation (i.e., completed using RStudio (RStudio Team, 2021) and the “rmcorr” R package) were performed using MATLAB R2021a (MathWorks, Inc., Natick, MA, USA).

## 3. Results

### 3.1. Summary of Data Collected

The sixteen female basketball athletes were prospectively monitored across two competitive collegiate basketball seasons, which consisted of (i) between two and three on-court sessions per week, (ii) one three-jump CMJ session per week, and (iii) weekly questionnaires which were completed once per week to obtain introspective, psychological state data. Fifteen athletes were monitored across the first competitive season, with one athlete discontinuing participation between the first and second seasons. Another athlete joined the team in the second season, resulting in a total of fifteen athletes monitored in season two; however, two of the athletes in season two discontinued their participation prior to the conclusion of the season, and thus, were only monitored for part of the season. Across the two competitive collegiate basketball seasons, a total of 1,226 on-court sessions were collected (mean per athlete = 77; SD per athlete = 26), 1,936 CMJ trials were performed (mean per athlete = 121; SD per athlete = 42), and 910 weekly self-reported questionnaires were completed (mean per athlete = 57; SD per athlete = 28). The average value and standard deviation for each outcome measure across all athletes stratified by season are presented in Table 1.

### 3.2. Biomechanical PCA Model Development

Given the highly correlated nature and redundancy of the included biomechanical metrics (Supplementary Table 1), a biomechanically focused PCA was trained using on-court and CMJ testing data obtained across two competitive seasons (i.e., 14 months of training). Initially, there were 865 weekly observations (i.e., on-court, CMJ, or self-reported introspective, psychological state data) amongst the cohort of 16 female basketball athletes. After filtering for weeks in which there were no missing biomechanical data, 394 synonymous weekly observations were identified and included in the PCA model (i.e., on-court and CMJ data collected during the same week of training; mean number of weeks that biomechanical data were collected per athlete = 25 (9)).

Following suggestions made by Bartholomew and Jolliffe et al. [57, 69], we retained n-PCs that explained ≥90% of the variance in our biomechanical data, which resulted in a total of eight extracted PCs (Table 2 and Supplementary Figure 1). The preseason reliability of the biomechanical PCs and the introspective, psychological state metrics are presented in Supplementary Table 2, while the correlations between the original biomechanical metrics and the newly derived PCs are presented in Supplementary Table 3.

While the loading coefficients for the biomechanical PCs represent complex relationships between the on-court and CMJ metrics used in the PCA, some general interpretations can be made of each biomechanical PC. Specifically, PC1 was loaded across a number of biomechanical metrics, signifying an “Overall Magnitude Component,” as is often the case for the first PC in a model. [73, 74] Specifically, higher scores in PC1 would relate to greater jump power and on-court impact intensity. Next, PC2 depicted an “On-Court Asymmetry Component,” reiterating the task-specific nature of biomechanical asymmetry [36–38]. Interestingly, PC1 (Overall Biomechanical Magnitude) and PC2 (On-Court Asymmetry) accounted for ∼50% of the variance in the data, suggesting the importance of both biomechanical constructs in our cohort. While PC3 was loaded with some on-court loading metrics, it was most heavily loaded with CMJ movement strategy metrics, signifying a “Jump Movement Strategy Component.” Next, PC4 was loaded with vertical jump braking power production and time-to-takeoff and on-court impact loading metrics, signifying an “On-Court Impact Loading Component” with interplay with the braking phase of vertical jump completion. PC5 was heavily loaded toward between-limb difference metrics from vertical jump testing and signified a “Jump Asymmetry Component.” While the relative importance of jump asymmetry was less than that of on-court asymmetry in our model (explained 7%, and 15% of the variance in the data, respectively), the composition of this PC further demonstrates the task specificity of biomechanical asymmetry. PC6 and PC7 presented similar, albeit inverse, loadings of initial biomechanical metrics, suggesting that on-court asymmetry in low-to medium-intensity bins may be associated with vertical jump power production. Lastly, PC8 was almost entirely related to the asymmetry that exists during the landing phase of movement during vertical jump testing and thus signifies a “Jump Landing-Phase Asymmetry Component.”

### 3.3. Association between Biomechanics and Introspective, Psychological State

Associations between biomechanical PCs and psychological state metrics were examined within each subject across the 2022-2023 season, which are summarized in this section and Supplementary Figures 2–6, with the longitudinal trends in these constructs highlighted in Supplementary Figures 7–11. It is important to note that even though data have been vastly reduced using the PCA (17 initial biomechanical variables to 5–8 PCs), there are still numerous relationships to examine in this context. Of these, the only relationships that reached statistical significance were pain with PC3 (i.e., jump movement strategy component; *r* = 0.19, *p* < 0.05) and with PC4 (i.e., on-court impact loading component; *r* = −0.18, *p* < 0.05) (Supplementary Figure 6). Otherwise, no overarching associations were identified at the group level between psychological state and biomechanical PCs. Nevertheless, it is important to note that these results depict significant in-phase relationships (i.e., does not account for possible phase-lagged relationships) and more temporally complex relationships which occur at the individual level may be missed. Therefore, the following section of the results will provide two use-case examples in our cohort of how such data could be used with calculated MDCs on an individual level to identify subject specific relationships.

### 3.4. Longitudinal PCA Use-Case and Application of Subject-Specific Monitoring Using Minimum Detectable Change Statistics

**Figure 1:**
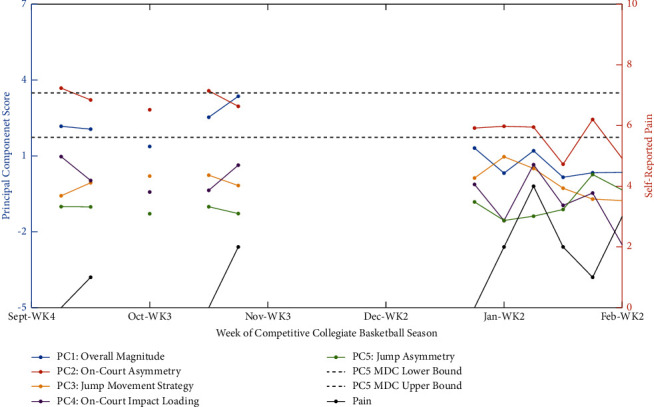
Use-case example of weekly changes in biomechanical principal component scores and self-reported pain in a collegiate female basketball athlete across the 2022-2023 competitive season. Specifically, on-court impact load and self-reported pain levels peaked before the detection of statistically significant alterations in on-court asymmetry. To highlight the on-court asymmetry-specific minimum detectable change (MDC), upper and lower bounds are depicted for red-flagging biomechanical fluctuations above and beyond the measurement error of the system. The MDC statistics are derived based on five-weeks of preseason training and normative biomechanical patterns exhibited at the cohort level, with this MDC value applied (±) to the average value that this subject displayed across the same timeframe to calculate individualized bounds by which their on-court asymmetry fluctuated from their normative patterns. It is important to note that this figure underrepresents the amount of data that was collected per participant over the two-year study period, as (i) only data from the 2022-2023 season are presented, as the first season (2021-2022) was only used to train the PCA model, and (ii) only weeks in which all three forms of data (i.e., on-court biomechanics, CMJ biomechanics, and psychological state) were concurrently collected are visually represented for simplicity sake and interpretability.

**Figure 2:**
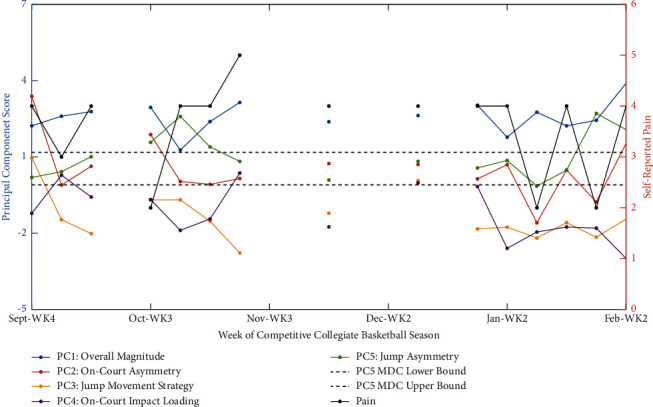
Use-case example of weekly changes in biomechanical principal component scores and self-reported pain in a collegiate female basketball athlete across the 2022-2023 competitive season. Specifically, jump asymmetry-specific minimum detectable change (MDC) upper and lower bounds are depicted for red-flagging biomechanical fluctuations above and beyond the measurement error of the system, which were paralleled, to some extent, by seasonal changes in self-reported levels of pain. The MDC statistics are derived based on five weeks of preseason training and normative biomechanical patterns exhibited at the cohort level, with this MDC value applied (±) to the average value that this subject displayed across the same timeframe to calculate individualized bounds by which their jump asymmetry fluctuated from their normative patterns. However, this “unperturbed” baseline period was confounded by high levels of self-reported pain in this athlete, suggesting that our normative biomechanical patterns might not necessarily indicate what we would expect in this athlete. It is important to note that this figure underrepresents the amount of data that was collected per participant over the two-year study period, as (i) only data from the 2022-2023 season are presented, as the first season (2021-2022) was only used to train the PCA model and (ii) only weeks in which all three forms of data (i.e., on-court biomechanics, CMJ biomechanics, and psychological state) were concurrently collected are visually represented for simplicity sake and interpretability.

To demonstrate the potential utility and challenges of incorporating PCs into athletic monitoring practices, Figures 1 and 2 highlight two case studies. Specifically, the first example (Figure 1) demonstrates an apparent connection between on-court asymmetry and impact loading related to pain. The athlete in the second example (Figure 2) was found to exhibit significant fluctuations in jump asymmetry, as well as pain across the season, but highlights the difficulty in establishing normative biomechanical patterns if the baseline period is confounded with elevated pain.

## 4. Discussion

This investigation was designed to determine the utility and application of longitudinal biopsychosocial athletic monitoring using a PCA model in collegiate female basketball players. We presented biomechanical and psychological state data longitudinally across two competitive seasons, highlighting seasonal changes in these constructs in our cohort. We found that biomechanical PCs were associated with pain throughout multiple competitive seasons but found that a cohort level approach limited the ability to detect more temporally complex (e.g., week-to-week) relationships and statistically or clinically relevant fluctuations from normative patterns. As such, using case studies, we highlighted the necessity to undertake an individualized and subject-specific approach to capture and delineate associations between biomechanics and introspective psychological states throughout a competitive season more adequately. Ours is the first study to integrate these performance-relevant domains using a biopsychosocial approach. Additionally, our investigation highlights the potential utility of prospectively and longitudinally monitoring student-athletes using a subject-specific approach and for integrating such models in future research and clinical work for sports performance, preventive, and prognostic purposes.

### 4.1. Seasonal Changes in Biomechanics and Introspective, Psychological State

There is a paucity of longitudinal investigations conducted in the realm of sports medicine [53], especially studies that have concurrently monitored and integrated biomechanics and psychology in a real-world setting using force plates and wearable technology. Reporting of seasonal data is seldom completed, and our study highlights within- and between-season variability that might be expected across multiple seasons in real-world and vertical jump biomechanics and self-reported psychological state. Generally, biomechanical and psychological state data were similar across seasons, aside from total impact load, total step count, and average intensity measured using IMUs during on-court basketball practices, which were found to be greater in the second season of our investigation. Meanwhile, the within-season variability in biomechanics exhibited in our study was consistent with previous work [20, 24, 75, 76]. It is important to note that this discrepancy between seasons might have been due to the one-month COVID-19-related shutdown during the first season, whereby our cohort of athletes had a significant reduction in training load both on- and off-court based on wearable and resistance-training (i.e., volume, intensity, and rate of perceived exertion) data, which never truly rebounded to preshutdown levels of training volume and intensity. Secondly, while most athletes (i.e., *n* = 14) returned to the team between seasons, the slight differences in our cohorts between seasons may have also contributed to the seasonal variability exhibited (Table 1). However, the within- and between-season variability exhibited in our study can begin to highlight the need for tailored and subject-specific approaches in athletic monitoring due to the inherent difficulty in measuring and interpreting these metrics with any level of consistency at the cohort level.

### 4.2. Association between Biomechanics and Introspective, Psychological State

We aimed to determine whether there were any overarching associations between biomechanics and psychology throughout a competitive collegiate basketball season. Our recent scoping review [53] found that pain and rating of perceived exertion were related to lower limb biomechanics and asymmetry. Unfortunately, these relationships that have been previously observed are highly variable in strength and rarely examined longitudinally [53]. While the findings of the current study support this association of biomechanics and pain, specifically with respect to jump movement strategy and on-court impact load (PC3 and PC4, respectively), the magnitude of these associations was weak (*r* = |0.18-0.19|, *p* < 0.05) [77], and there were no other clear or overarching associations identified at the cohort level when controlling for between-subject variability.

Our results suggest that the overlap between domains is, at best, limited at the group level, highlighting the necessity to undertake an individualized and subject-specific approach. Moreover, trying to establish contributory factors to sports performance and risk of reinjury at the group level is something that may not be possible or even sensible to do in many cases. Similar subject-specific machine learning model suggestions to identify biomechanical patterns and detect alterations have been made in runners [78, 79] and patients diagnosed with knee osteoarthritis [80]. A similar approach in sports medicine and athletic monitoring may enable practitioners to reduce the injury burden, which can be quite substantial [4, 5]. We theorized that developing subject-specific models and associated MDC thresholds in athletic populations can be of use for decision support systems where an athlete may be red-flagged for further analysis to ultimately improve preventive medical practices. In other words, these individualized biomechanical patterns can signify (i) a “green-light” with no sign of concern when individuals display normative or unchanging biomechanical patterns, (ii) changes outside or nearing outside of this MDC threshold might signify a “yellow (caution)-light” where some concern is present and follow-up may be required, and (iii) significant biomechanical alterations from normal patterns signifying a “red-light” which might be indicative of larger concern and an immediate and further investigation of the athlete's current condition.

### 4.3. Longitudinal PCA Use-Case and Application of Minimum Detectable Change Statistics for Red-Flagging Athletes

Two case examples highlighted the longitudinal monitoring using PCs and associated MDCs to identify alterations from normative biomechanical patterns on a subject-specific basis. The first example identified fluctuations in on-court asymmetry patterns and concurrent or prior changes in perceived pain and on-court impact load (Figure 1). In contrast, the second example focuses on identifying alterations in jump asymmetry patterns while highlighting the potential difficulty in establishing normative biomechanical patterns without contextualizing them with other facets of health, such as self-reported pain levels (Figure 2).

In the first example (Figure 1), it is apparent that this female athlete showed fluctuations beyond their typical on-court asymmetry patterns near the end of the 2022-2023 competitive basketball season. Seven months prior, during the preseason, this individual displayed consistent PC2 scores near values of 3, which can be contextualized here as being left limb dominant (−18.1% between-limb difference in favour of the left limb) during high-intensity aspects of practices. However, in early 2023, the same metric (PC2) was near the boundary of the MDC (e.g., value of 2.1), relating to a −10.5% between-limb difference, favouring the left limb in high-intensity aspects of practice. Most importantly, this PC2 deviation was also met with a gradual increase in pain towards an eventual peak in the third week of January. Consequently, while the athlete was nearing a red-flag scenario before this elevated pain, they exceeded their normal pattern of on-court asymmetry in the week following peak pain onset. The manifestation of this was a PC2 score of 0.7 and a −0.5% between-limb difference (i.e., no between limb difference during high-intensity aspects of these sessions). In other words, this athlete was someone that generally displayed a left dominant pattern in high-intensity aspects of practice but had now begun to alter their pattern to reduce the loading on that left limb. The medical staff chart notes supported that the timing of this deviation coincided with lower limb MSK soreness and tightness which required ongoing management throughout the remainder of the season, even though no specific injury or time loss occurred. It is important to note that while this moved them into a more symmetrical pattern relative to their previous baseline biomechanical pattern, this was a clinically meaningful deviation [16–23], to an extent (10–15%) that has at times been related to heightened injury risk [18, 24–27]. Interestingly, this fluctuation from normative asymmetry patterns appeared most pronounced during high-intensity efforts. This observation is concerning as most serious lower limb MSK injuries (e.g., anterior cruciate ligament rupture) are sustained during high-intensity landings from bouts of jumping, during a change-of-direction, or cutting maneuvers in basketball [1, 9–11]. Moreover, in the weeks following this red-flag situation, the athlete had various additional biomechanical changes. Most notably, the successive reduction of on-court loading (PC4) highlights a possible attempt to manage their pain and potential underlying injury. Generally, exceeding MSK structure load tolerance during training and competition can lead to injury [1, 9–11], and this may be exacerbated if there are large between-limb differences that exist and one limb is unduly stressed [18, 24–27] or, in this case, stressed the contralateral (right) limb more than was previously typical. As such, athletes will often attempt to restore and maintain homeostasis and mitigate the underlying cause of pain by reducing the load and frequency of the activities leading to an injury [81], which appears to be the case for this athlete, evident in not only on-court loading (PC4) but also jump movement strategy (PC3). Nevertheless, it is important to note that this is a single-case study and interpretation of these changes occurring.

The second case example (Figure 2) highlighted seasonal fluctuations (i) during the end of the preseason in October 2022 and (ii) after the regular season just before collegiate-level playoffs in February 2023 that exceeded the MDC threshold in normative jump asymmetry patterns that we were able to red-flag for further examination. When observing the force-time waveforms and associated jump asymmetry metrics that were heavily weighted in PC5, it was found that peak braking force asymmetry, peak propulsive force asymmetry, and average braking rate of force development asymmetry had all fluctuated from normative patterns during the final week in October (i.e., Δ from mean = 8–12%), with both peak braking and propulsive force asymmetry also demonstrating changes in right-to-left limb dominance. While the fluctuations this athlete exhibited in jump asymmetry exceeded the MDC thresholds for statistical relevance, none exceeded the 10–15% threshold for clinical relevance [18, 82]; thus, there was not a serious or urgent (i.e., red-flag) concern but rather an indication to proceed with caution in regards to this athlete. Interestingly, the on-court asymmetry and self-reported pain from this athlete, as seen in orange and black, respectively, in Figure 2, displayed similar and somewhat paralleled fluctuations to jump asymmetry. However, it is noteworthy that this athlete was experiencing moderate levels of pain during the preseason and minor overuse injuries during competitive season, as noted by the sport medicine team. This pain information was used to define normative biomechanical patterns for this individual and for building out the associated MDC values. While these minor injuries did not force the athlete to miss any practice or competitions, they did necessitate continuous management to address tightness and soreness related to the MSK issues. As such, these fluctuations identified as red-flags might not necessarily indicate meaningful fluctuations from this individual's normative biomechanical patterns since the predefined baseline period was not pain-free and thus not normal for this individual. Additionally, this demonstrates the high levels of variability in biomechanics and prevalence of pain that may exist in athletic populations across a season without an injury that constitutes time loss. This is in line with other work that has discussed the variability in pain tolerance and athletes' coping mechanisms to mitigate such pain in athletic populations [83, 84]. Cumulatively, this example highlights a potential limitation of this PCA model since the MDC values should ideally be established during a stable, pain-free period; however, such stability cannot be guaranteed to exist for all athletes in this cohort nor can the potential parallels between jump asymmetry and perceived pain. However, this case provides a practical example of how PCA and this biopsychosocial model can be applied in longitudinal contexts for potential preventive and prognostic purposes while highlighting considerations that need to be made when employing such models.

### 4.4. Limitations and Future Directions

There are several limitations to the present investigation, many of which have been previously outlined in a similar longitudinal vertical jump PCA application of ours [63] and a corresponding master's thesis document [85]. First, this sample consisted of a homogenous group of female collegiate basketball athletes and, thus, may limit the generalizability to males or other competitive athletic populations given that previous PCA work has demonstrated that the makeup of these models can differ between sports [86–88] and based on biological sex [87, 89]. Similarly, despite the limited size of our sample, the numerous repeated observations and the comprehensive visualizations of the data instill confidence in the relationships (or lack thereof) presented here. However, it is important to acknowledge that while we believe our study effectively characterizes the relationships within our current sample, these findings may not necessarily apply to other athlete cohorts or populations. Similarly, while the PCs and associated MDCs from the current work are specific to this cohort and dataset, the reliability of this method and the practical use-case examples should provide confidence in using such methods in future research and clinical applications. Additionally, it is possible that the effects of the menstrual cycle may have affected jump and on-court biomechanics due to potential alterations in neuromuscular function [90]; however, recent systematic reviews have suggested that the effects of the menstrual cycle on exercise performance are inconclusive and trivial [91, 92]. Second, the associations identified between biomechanical and psychological states were established across all data rather than assessing the association between weekly changes in these constructs. Future research should examine the temporal association between biomechanics and psychological state using cross-correlation to discern whether changes in one entity precede the other. Third, while this PCA model includes both on-court and traditional CMJ biomechanics, we did not include our psychological state metrics in this model due to the low compliance rates exhibited in the first year of our investigation (compliance rates in season one: academic workload = 67 ± 16%; feeling = 80 ± 11%; sleep = 55 ± 29%; pain = 49 ± 29%). The potential implications of excluding psychological state metrics from our PCA model remain relatively unknown, as many of our included psychological state metrics have not been evaluated in conjunction with lower limb biomechanics, and the relationship between these constructs has, to our knowledge, not been elucidated in a longitudinal setting. Therefore, it is suggested that future research examines the utility in integrating psychological state into their PCA models to define multivariate and multilevel PCs that capture a greater degree of the athlete's status. Fourth, the PCA model in the present investigation was built on variation in data between athletes rather than developing numerous potentially more sensitive subject-specific models. Unfortunately, training a multivariate, subject-specific PCA model would require many jumps and on-court sessions (e.g., 50 or more per athlete) and result in similar but unique PC profiles across athletes that would require separate interpretations. Since the overarching purpose and proposed utility of the PCA model and the associated MDCs in the present investigation was for red-flagging athletes, this might not be necessary, and doing so may cause more difficulty and time constraints in this process. However, future work could investigate whether subject-specific models are more appropriate for detecting more subtle individual changes from normative patterns than the present between-subject model.

## 5. Conclusion and Perspective

Our study is the first to use advanced analytical modeling to characterize components of student-athlete performance and well-being and the only study to integrate these domains in a longitudinal and biopsychosocial fashion over multiple competitive seasons. Our data showed associations and overlap between biomechanical and psychological patterns in athletic populations and emphasized the highly subject-specific nature of these associations. We propose that there is a need for more tailored and subject-specific athletic monitoring practices, particularly those that include integrated, as opposed to traditionally isolated, biomechanical, physiological, and psychological athletic monitoring. Specifically, we showed the ability to incorporate this methodological approach for prospectively red-flagging athletes who demonstrate statistically and clinically relevant fluctuations in normative biomechanical and psychological patterns that might indicate a heightened risk of injury or decrements in sports performance. Future research should employ similar biopsychosocial PCA models to prospectively monitor athletic populations to determine the potential preventive and prognostic capabilities of athletic monitoring practices.

## Figures and Tables

**Table 1:** Descriptive statistics of biomechanical and psychological state metrics stratified by study year.

Measure	Study year 2021-2022		Study year 2022-2023	
	Mean	SD	Mean	SD
Total impact load (sum of steps at each intensity in g's)	72,150	25,766	83,873	33,751
Total step count	6004	1199	6705	1549
Ave. intensity (g)	11.83	2.72	12.30	3.37
Impact Asym. (%)	4.32	3.36	3.77	3.36
Low-G Asym. (%)	2.02	2.0	1.86	1.57
Medium-G Asym. (%)	3.77	2.86	3.13	2.60
High-G Asym. (%)	7.27	5.44	6.57	5.97
Jump height (m)	0.24	0.05	0.26	0.05
CMD (m)	−0.28	0.04	−0.28	0.04
Time to takeoff (s)	0.84	0.11	0.79	0.10
Pk Rel. Brk power (W/kg)	−13.84	3.19	−14.51	2.99
Pk Rel. Prop power (W/kg)	39.90	5.03	41.91	5.74
Pk Brk force Asym. (%)	5.58	4.88	5.43	4.12
Pk Prop force Asym. (%)	3.74	3.11	3.70	3.07
Ave. Brk RFD Asym. (%)	9.69	8.03	8.81	6.53
Pk Lnd force Asym. (%)	11.24	9.34	9.70	10.17
RSI Mod. (JH/contact time)	0.29	0.06	0.33	0.08
Academic workload (0–10)	5.85	1.84	5.28	2.38
Feeling (0–10)	6.36	1.52	5.88	1.60
Sleep quantity–PSQI component 3 (0–3)	N/A	N/A	0.58	0.74
Sleep quality–PSQI component 1 (0–3)	N/A	N/A	0.99	0.75
Pain (0–10)	2.99	1.50	1.36	1.69

SD = standard deviation; Ave. = average; Asym. = asymmetry; CMD = countermovement depth; Pk = peak; Rel. = relative; Brk = braking; Prop = propulsive; RFD = rate of force development; Lnd = landing; RSI Mod. = the modified reactive strength index; PSQI = Pittsburgh sleep quality index. *Note.* Asymmetry metrics computed as absolute values to demonstrate the within- and between-season variability in the magnitude of asymmetry across a team but is blinded to the nuances of fluctuations in limb dominance in doing so. Sleep quantity and quality collected too inconsistently in season one to report and provide between-season comparisons. Higher scores in feeling represent better mental health, while higher scores in academic workload and pain represent a greater workload and level of perceived pain, respectively. Lower scores on sleep scales represent greater sleep quality and quantity.

**Table 2:** Summary of principal component analysis loading coefficients.

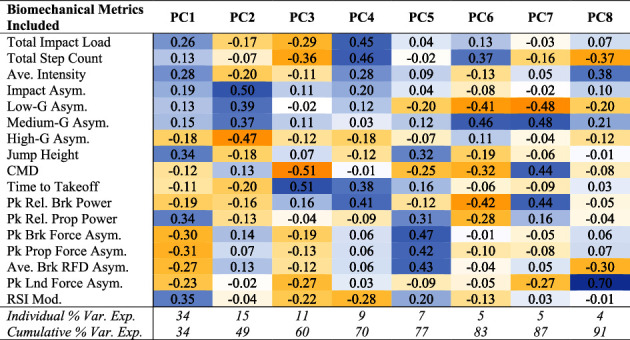

PC = principal component; Ave. = average; Asym. = asymmetry; CMD = countermovement depth; Pk = peak; Rel. = relative; Brk = braking; Prop = propulsive; RFD = rate of force development; Lnd = landing; RSI Mod. = the modified reactive strength index; % Var. Exp. = percent variance explained. The magnitude and direction of the relationships found are indicated using a colour-coded scale, such that the relationships become increasingly more negative with darker shades of yellow, while the relationships become increasingly more positive with deeper shades of blue.

## Data Availability

The datasets generated and/or analyzed during the current study are available from the corresponding author on reasonable request.

## Supplementary Materials

Supplementary Materials Supplementary Table 1: correlations between on-court inertial measurement unit-derived and force plate-derived countermovement jump biomechanical metrics in a cohort of collegiate female basketball athletes from data across two competitive seasons. The magnitude and direction of the relationships found are indicated using a colour-coded scale, such that the relationships become increasingly more negative with darker shades of yellow, while the relationships become increasingly more positive with deeper shades of blue. Supplementary Table 2: the reliability of biomechanical principal component scores and psychological state metrics collected across five weeks of the 2022-2023 competitive, collegiate female basketball preseason, along with the corresponding standard error of the measurement and minimum detectable change statistics. Supplementary Table 3: correlation between original biomechanical variables and newly derived principal component scores. The magnitude and direction of the relationships found are indicated using a colour-coded scale, such that the relationships become increasingly more negative with darker shades of yellow, while the relationships become increasingly more positive with deeper shades of blue. Supplementary Figure 1: scree plot of log-eigenvalues of each principal component in the biomechanical PCA model vs. the number of principal components in the model. Supplementary Figure 2: repeated measures correlation-based associations identified between biomechanical principal component scores and self-reported academic workload across the 2022-2023 season, with subject-specific data distinguished using different colours, and the commonality in within-individual associations after controlling for between-individual variance identified in the bottom right corner of each subplot. Supplementary Figure 3: repeated measures correlation-based associations identified between biomechanical principal component scores and self-reported feeling across the 2022-2023 season, with subject-specific data distinguished using different colours, and the commonality in within-individual associations after controlling for between-individual variance identified in the bottom right corner of each subplot. Supplementary Figure 4: repeated measures correlation-based associations identified between biomechanical principal component scores and self-reported sleep quantity across the 2022-2023 season, with subject-specific data distinguished using different colours, and the commonality in within-individual associations after controlling for between-individual variance identified in the bottom right corner of each subplot. Supplementary Figure 5: repeated measures correlation-based associations identified between biomechanical principal component scores and self-reported sleep quality across the 2022-2023 season, with subject-specific data distinguished using different colours, and the commonality in within-individual associations after controlling for between-individual variance identified in the bottom right corner of each subplot. Supplementary Figure 6: repeated measures correlation-based associations identified between biomechanical principal component scores and self-reported levels of pain across the 2022-2023 season, with subject-specific data distinguished using different colours, and the commonality in within-individual associations after controlling for between-individual variance identified in the bottom right corner of each subplot. Supplementary Figure 7: weekly changes in biomechanical principal component scores and self-reported academic workload in a cohort of collegiate female basketball athletes across the 2022-2023 competitive season. Supplementary Figure 8: weekly changes in biomechanical principal component scores and self-reported feeling in a cohort of collegiate female basketball athletes across the 2022-2023 competitive season. Supplementary Figure 9: weekly changes in biomechanical principal component scores and self-reported sleep quantity in a cohort of collegiate female basketball athletes across the 2022-2023 competitive season. Supplementary Figure 10: weekly changes in biomechanical principal component scores and self-reported sleep quality in a cohort of collegiate female basketball athletes across the 2022-2023 competitive season. Supplementary Figure 11: weekly changes in biomechanical principal component scores and self-reported pain in a cohort of collegiate female basketball athletes across the 2022-2023 competitive season.
