# Supplementary material for: Longitudinal Monitoring of Biomechanical and Psychological State in Collegiate Female Basketball Athletes Using Principal Component Analysis
Source: Transl Sports Med. 2024 Apr 3;2024:7858835. doi: 10.1155/2024/7858835 (PMC11023736; doi:10.1155/2024/7858835)
Supplement: Supplementary Materials — Supplementary Table 1: correlations between on-court inertial measurement unit-derived and force plate-derived countermovement jump biomechanical metrics in a cohort of collegiate female basketball athletes from data across two competitive seasons. The magnitude and direction of the relationships found are indicated using a colour-coded scale, such that the relationships become increasingly more negative with darker shades of yellow, while the relationships become increasingly more positive with deeper shades of blue. Supplementary Table 2: the reliability of biomechanical principal component scores and psychological state metrics collected across five weeks of the 2022-2023 competitive, collegiate female basketball preseason, along with the corresponding standard error of the measurement and minimum detectable change statistics. Supplementary Table 3: correlation between original biomechanical variables and newly derived principal component scores. The magnitude and direction of the relationships found are indicated using a colour-coded scale, such that the relationships become increasingly more negative with darker shades of yellow, while the relationships become increasingly more positive with deeper shades of blue. Supplementary Figure 1: scree plot of log-eigenvalues of each principal component in the biomechanical PCA model vs. the number of principal components in the model. Supplementary Figure 2: repeated measures correlation-based associations identified between biomechanical principal component scores and self-reported academic workload across the 2022-2023 season, with subject-specific data distinguished using different colours, and the commonality in within-individual associations after controlling for between-individual variance identified in the bottom right corner of each subplot. Supplementary Figure 3: repeated measures correlation-based associations identified between biomechanical principal component scores and self-reported feeling across the 20 [file 7858835.f1.docx]

**Supplementary Table 1.** Correlations between on-court inertial measurement unit-derived and force plate-derived countermovement jump biomechanical metrics in a cohort of collegiate female basketball athletes from data across two competitive seasons. The magnitude and direction of the relationships found are indicated using a colour coded scale, such that the relationships become increasingly more negative with darker shades of yellow, while the relationships become increasingly more positive with deeper shades of blue.

|  | **TIL** | **TSC** | **Ave Int** | **Impact Asym** | **Low-G Asym** | **Medium-G Asym** | **High-G Asym** | **JH** | **CMD** | **TTTo** | **PRBP** | **PRPP** | **PBF Asym** | **PPF Asym** | **Ave BRFD Asym** | **PLF Asym** | **RSI Mod** |
| --- | --- | --- | --- | --- | --- | --- | --- | --- | --- | --- | --- | --- | --- | --- | --- | --- | --- |
| **TIL** | 1.00 (1.00, 1.00) | **0.75 (0.70, 0.79) ***** | **0.83 (0.80, 0.86) ***** | **0.14 (0.04, 0.23) **** | 0.08 (-0.02, 0.18) | 0.08 (-0.02, 0.18) | **-0.11 (-0.21, -0.01) *** | **0.46 (0.38, 0.54) ***** | -0.04 (-0.14, 0.06) | **-0.11 (-0.21, -0.01) *** | **-0.12 (-0.22, -0.02) *** | **0.50 (0.42, 0.57) ***** | **-0.33 (-0.42, -0.24) ***** | **-0.36 (-0.45, -0.27) ***** | **-0.36 (-0.44, -0.26) ***** | **-0.18 (-0.27, -0.08) ***** | **0.47 (0.39, 0.55) ***** |
| **TSC** | **0.75 (0.70, 0.79) ***** | 1.00 (1.00, 1.00) | **0.28 (0.19, 0.37) ***** | 0.08 (-0.03, 0.17) | 0.07 (-0.03, 0.17) | 0.05 (-0.05, 0.15) | -0.05 (-0.15, 0.05) | **0.16 (0.06, 0.25) **** | 0.10 (0.00, 0.20) | **-0.15 (-0.24, -0.05) **** | **-0.10 (-0.20, 0.00) *** | **0.19 (0.09, 0.28) ***** | **-0.11 (-0.21, -0.01) *** | **-0.17 (-0.27, -0.07) ***** | -0.08 (-0.18, 0.02) | -0.05 (-0.15, 0.05) | **0.22 (0.12, 0.31) ***** |
| **Ave Int** | **0.83 (0.80, 0.86) ***** | **0.28 (0.19, 0.37) ***** | 1.00 (1.00, 1.00) | **0.13 (0.03, 0.23) *** | 0.07 (-0.03, 0.17) | 0.04 (-0.06, 0.14) | **-0.12 (-0.22, -0.02) *** | **0.56 (0.49, 0.63) ***** | **-0.17 (-0.26, -0.07) **** | -0.02 (-0.12, 0.08) | -0.09 (-0.19, 0.01) | **0.58 (0.50, 0.64) ***** | **-0.39 (-0.48, -0.31) ***** | **-0.38 (-0.46, -0.29) ***** | **-0.46 (-0.53, -0.37) ***** | **-0.24 (-0.33, -0.14) ***** | **0.50 (0.42, 0.57) ***** |
| **Impact Asym** | **0.14 (0.04, 0.23) **** | 0.08 (-0.03, 0.17) | **0.13 (0.03, 0.23) *** | 1.00 (1.00, 1.00) | **0.65 (0.59, 0.71) ***** | **0.62 (0.55, 0.68) ***** | **-0.87 (-0.89, -0.85) ***** | **0.16 (0.06, 0.26) **** | -0.06 (-0.16, 0.04) | **-0.13 (-0.23, -0.03) **** | **-0.25 (-0.34, -0.15) ***** | **0.20 (0.10, 0.29) ***** | **-0.15 (-0.25, -0.06) **** | **-0.23 (-0.32, -0.13) ***** | **-0.13 (-0.23, -0.03) **** | **-0.26 (-0.35, -0.16) ***** | **0.22 (0.12, 0.31) ***** |
| **Low-G Asym** | 0.08 (-0.02, 0.18) | 0.07 (-0.03, 0.17) | 0.07 (-0.03, 0.17) | **0.65 (0.59, 0.71) ***** | 1.00 (1.00, 1.00) | **0.13 (0.03, 0.23) **** | **-0.55 (-0.62, -0.48) ***** | 0.08 (-0.03, 0.17) | 0.07 (-0.03, 0.17) | **-0.22 (-0.32, -0.13) ***** | **-0.20 (-0.29, -0.10) ***** | 0.10 (0.00, 0.20) | **-0.16 (-0.25, -0.06) **** | **-0.18 (-0.28, -0.08) ***** | **-0.14 (-0.24, -0.04) **** | **-0.15 (-0.25, -0.05) **** | **0.18 (0.08, 0.27) ***** |
| **Medium-G Asym** | 0.08 (-0.02, 0.18) | 0.05 (-0.05, 0.15) | 0.04 (-0.06, 0.14) | **0.62 (0.55, 0.68) ***** | **0.13 (0.03, 0.23) **** | 1.00 (1.00, 1.00) | **-0.56 (-0.63, -0.49) ***** | 0.08 (-0.02, 0.18) | **-0.11 (-0.21, -0.01) *** | **-0.19 (-0.28, -0.09) ***** | **-0.29 (-0.38, -0.19) ***** | **0.16 (0.06, 0.25) **** | **-0.11 (-0.21, 0.01) *** | **-0.22 (-0.31, -0.12) ***** | **-0.11 (-0.21, 0.01) *** | **-0.27 (-0.36, -0.18) ***** | **0.19 (0.09, 0.28) ***** |
| **High-G Asym** | **-0.11 (-0.21, -0.01) *** | -0.05 (-0.15, 0.05) | **-0.12 (-0.22, -0.02) *** | **-0.87 (-0.89, -0.85) ***** | **-0.55 (-0.62, -0.48) ***** | **-0.56 (-0.63, -0.49) ***** | 1.00 (1.00, 1.00) | **-0.18 (-0.27, -0.08) ***** | 0.05 (-0.05, 0.15) | **0.12 (0.01, 0.21) *** | **0.21 (0.11, 0.30) ***** | **-0.24 (-0.33, -0.14) ***** | **0.14 (0.04, 0.24) **** | **0.23 (0.13, 0.32) ***** | **0.14 (0.04, 0.23) **** | **0.26 (0.16, 0.35) ***** | **-0.22 (-0.31, -0.12) ***** |
| **JH** | **0.46 (0.38, 0.54) ***** | **0.16 (0.06, 0.25) **** | **0.56 (0.49, 0.63) ***** | **0.16 (0.06, 0.26) **** | 0.08 (-0.03, 0.17) | 0.08 (-0.02, 0.18) | **-0.18 (-0.27, -0.08) ***** | 1.00 (1.00, 1.00) | **-0.43 (-0.51, -0.34) ***** | -0.01 (-0.11, 0.09) | **-0.32 (-0.41, -0.23) ***** | **0.94 (0.93, 0.95) ***** | **-0.51 (-0.58, -0.44) ***** | **-0.50 (-0.57, -0.42) ***** | **-0.46 (-0.54, -0.38) ***** | **-0.43 (-0.51, -0.35) ***** | **0.85 (0.82, 0.87) ***** |
| **CMD** | -0.04 (-0.14, 0.06) | 0.10 (-0.00, 0.20) | **-0.17 (-0.26, -0.07) **** | -0.06 (-0.16, 0.04) | 0.07 (-0.03, 0.17) | **-0.11 (-0.21, -0.01) *** | 0.05 (-0.05, 0.15) | **-0.43 (-0.51, -0.34) ***** | 1.00 (1.00, 1.00) | **-0.50 (-0.57, -0.42) ***** | **0.21 (0.11, 0.31) ***** | **-0.21 (-0.30, -0.11) ***** | **0.27 (0.18, 0.36) ***** | **0.21 (0.11, 0.30) ***** | **0.25 (0.16, 0.35) ***** | **0.32 (0.23, 0.41) ***** | -0.08 (-0.18, -0.02) |
| **TTTo** | **-0.11 (-0.21, -0.01) *** | **-0.15 (-0.24, -0.05) **** | 0.02 (-0.12, 0.08) | **-0.13 (-0.23, -0.03) **** | **-0.22 (-0.32, -0.13) ***** | **-0.19 (-0.28, -0.09) ***** | **0.12 (0.01, 0.21) *** | 0.01 (-0.11, 0.09) | **-0.50 (-0.57, -0.42) ***** | 1.00 (1.00, 1.00) | **0.53 (0.45, 0.60) ***** | **-0.13 (-0.23, -0.03) *** | 0.06 (-0.04, 0.16) | **0.13 (0.03, 0.23) *** | **0.12 (0.02, 0.21) *** | -0.03 (-0.13, 0.07) | **-0.51 (-0.58, -0.43) ***** |
| **PRBP** | **-0.12 (-0.22, -0.02) *** | **-0.10 (-0.20, 0.00) *** | 0.09 (-0.19, 0.01) | **-0.25 (-0.34, -0.15) ***** | **-0.20 (-0.29, -0.10) ***** | **-0.29 (-0.38, -0.19) ***** | **0.21 (0.11, 0.30) ***** | **-0.32 (-0.41, -0.23) ***** | **0.21 (0.11, 0.31) ***** | **0.53 (0.45, 0.60) ***** | 1.00 (1.00, 1.00) | **-0.24 (-0.33, -0.14) ***** | **0.17 (0.07, 0.27) ***** | **0.28 (0.18, 0.37) ***** | **0.22 (0.12, 0.32) ***** | **0.16 (0.06, 0.26) **** | **-0.55 (-0.61, -0.47) ***** |
| **PRPP** | **0.50 (0.42, 0.57) ***** | **0.19 (0.09, 0.28) ***** | **0.58 (0.50, 0.64) ***** | **0.20 (0.10, 0.29) ***** | 0.10 (0.00, 0.20) | **0.16 (0.06, 0.25) **** | **-0.24 (-0.33, -0.14) ***** | **0.94 (0.93, 0.95) ***** | **-0.21 (-0.30, -0.11) ***** | **-0.13 (-0.23, -0.03) *** | **-0.24 (-0.33, -0.14) ***** | 1.00 (1.00, 1.00) | **-0.48 (-0.55, -0.40) ***** | **-0.49 (-0.56, -0.41) ***** | **-0.40 (-0.48, -0.31) ***** | **-0.43 (-0.51, -0.34) ***** | **0.87 (0.84, 0.89) ***** |
| **PBF Asym** | **-0.33 (-0.42, -0.24) ***** | **-0.11 (-0.21, -0.01) *** | **-0.39 (-0.48, -0.31) ***** | **-0.15 (-0.25, -0.06) **** | **-0.16 (-0.25, -0.06) **** | **-0.11 (-0.21, 0.01) *** | **0.14 (0.04, 0.24) **** | **-0.51 (-0.58, -0.44) ***** | **0.27 (0.18, 0.36) ***** | 0.06 (-0.04, 0.16) | **0.17 (0.07, 0.27) ***** | **-0.48 (-0.55, -0.40) ***** | 1.00 (1.00, 1.00) | **0.87 (0.84, 0.89) ***** | **0.73 (0.68, 0.78) ***** | **0.43 (0.34, 0.51) ***** | **-0.46 (-0.53, -0.37) ***** |
| **PPF Asym** | **-0.36 (-0.45, -0.27) ***** | **-0.17 (-0.27, -0.07) ***** | **-0.38 (-0.46, -0.29) ***** | **-0.23 (-0.32, -0.13) ***** | **-0.18 (-0.28, -0.08) ***** | **-0.22 (-0.31, -0.12) ***** | **0.23 (0.13, 0.32) ***** | **-0.50 (-0.57, -0.42) ***** | **0.21 (0.11, 0.30) ***** | **0.13 (0.03, 0.23) *** | **0.28 (0.18, 0.37) ***** | **-0.49 (-0.56, -0.41) ***** | **0.87 (0.84, 0.89) ***** | 1.00 (1.00, 1.00) | **0.65 (0.58, 0.70) ***** | **0.41 (0.32, 0.49) ***** | **-0.49 (-0.56, -0.41) ***** |
| **Ave BRFD Asym** | **-0.36 (-0.44, -0.26) ***** | -0.08 (-0.18, 0.02) | **-0.46 (-0.53, -0.37) ***** | **-0.13 (-0.23, -0.03) **** | **-0.14 (-0.24, -0.04) **** | **-0.11 (-0.21, 0.01) *** | **0.14 (0.04, 0.23) **** | **-0.46 (-0.54, -0.38) ***** | **0.25 (0.16, 0.35) ***** | **0.12 (0.02, 0.21) *** | **0.22 (0.12, 0.32) ***** | **-0.40 (-0.48, -0.31) ***** | **0.73 (0.68, 0.78) ***** | **0.65 (0.58, 0.70) ***** | 1.00 (1.00, 1.00) | **0.29 (0.20, 0.38) ***** | **-0.44 (-0.52, -0.36) ***** |
| **PLF Asym** | **-0.18 (-0.27, -0.08) ***** | -0.05 (-0.15, 0.05) | **-0.24 (-0.33, -0.14) ***** | **-0.26 (-0.35, -0.16) ***** | **-0.15 (-0.25, -0.05) **** | **-0.27 (-0.36, -0.18) ***** | **0.26 (0.16, 0.35) ***** | **-0.43 (-0.51, -0.35) ***** | **0.32 (0.23, 0.41) ***** | -0.03 (-0.13, 0.07) | **0.16 (0.06, 0.26) **** | **-0.43 (-0.51, -0.34) ***** | **0.43 (0.34, 0.51) ***** | **0.41 (0.32, 0.49) ***** | **0.29 (0.20, 0.38) ***** | 1.00 (1.00, 1.00) | **-0.36 (-0.44, -0.27) ***** |
| **RSI Mod** | **0.47 (0.39, 0.55) ***** | **0.22 (0.12, 0.31) ***** | **0.50 (0.42, 0.57) ***** | **0.22 (0.12, 0.31) ***** | **0.18 (0.08, 0.27) ***** | **0.19 (0.09, 0.28) ***** | **-0.22 (-0.31, -0.12) ***** | **0.85 (0.82, 0.87) ***** | -0.08 (-0.18, -0.02) | **-0.51 (-0.58, -0.43) ***** | **-0.55 (-0.61, -0.47) ***** | **0.87 (0.84, 0.89) ***** | **-0.46 (-0.53, -0.37) ***** | **-0.49 (-0.56, -0.41) ***** | **-0.44 (-0.52, -0.36) ***** | **-0.36 (-0.44, -0.27) ***** | 1.00 (1.00, 1.00) |
| Abbreviations: TIL = on-court total impact load; TSC = on-court total step count; Ave Int = on-court average intensity; Impact Asym = on-court impact asymmetry; Low-G Asym = on-court low-intensity impact asymmetry; Medium-G Asym = on-court medium-intensity impact asymmetry; High-G Asym = on-court high-intensity impact asymmetry; JH = jump height; CMD = countermovement depth; TTTo = time to takeoff; PRBP = peak relative braking power; PRPP = peak relative propulsive power; PBF Asym = peak braking force asymmetry; PPF Asym = peak propulsive force asymmetry; Ave BRFD Asym = average braking rate of force development asymmetry; PLF Asym = peak landing force asymmetry; RSI mod = the modified reactive strength index; * = p<0.05; ** = p<0.01; *** = p<0.001. | | | | | | | | | | | | | | | | | |

**Supplementary Table 2.** The reliability of biomechanical principal component scores and psychological state metrics collected across five-weeks of the 2022-2023 competitive, collegiate female basketball preseason, along with the corresponding standard error of the measurement and minimum detectable change statistics.

| **PCs and Psychological State Metrics** | **Mean (SD)** | **ICC** | **95% CI** | **SEM** | **MDC** |
| --- | --- | --- | --- | --- | --- |
| PC1 | 0.41 (2.22) | 1.00 | 0.99 - 1.00 | 0.15 | 0.42 |
| PC2 | -0.29 (1.46) | 0.95 | 0.90 - 0.98 | 0.32 | 0.88 |
| PC3 | -0.75 (1.18) | 0.93 | 0.85 - 0.97 | 0.32 | 0.89 |
| PC4 | 0.59 (0.97) | 0.91 | 0.81 - 0.97 | 0.29 | 0.81 |
| PC5 | 0.07 (1.07) | 0.95 | 0.89 - 0.98 | 0.23 | 0.64 |
| PC6 | 0.28 (0.92) | 0.92 | 0.83 - 0.97 | 0.27 | 0.74 |
| PC7 | -0.25 (0.84) | 0.93 | 0.83 - 0.98 | 0.22 | 0.60 |
| PC8 | -0.38 (0.83) | 0.91 | 0.81 - 0.97 | 0.25 | 0.69 |
| Academic Workload | 5.25 (1.90) | 0.71 | 0.41 - 0.89 | 1.02 | 2.83 |
| Feeling | 6.15 (1.63) | 0.77 | 0.52 - 0.91 | 0.78 | 2.16 |
| Sleep Quantity | 0.66 (0.86) | 0.72 | 0.42 - 0.89 | 0.46 | 1.26 |
| Sleep Quality | 1.01 (0.74) | 0.89 | 0.77 - 0.96 | 0.24 | 0.68 |
| Pain | 1 (1.29) | 0.89 | 0.76 - 0.96 | 0.44 | 1.21 |
| Abbreviations: PC = principal component; SD = standard deviation; ICC = intraclass correlation coefficient; CI = confidence interval; SEM = standard error of the measurement; MDC = minimum detectable change. | | | | | |

**Supplementary Table 3.** Correlation between original biomechanical variables and newly derived principal component scores. The magnitude and direction of the relationships found are indicated using a colour coded scale, such that the relationships become increasingly more negative with darker shades of yellow, while the relationships become increasingly more positive with deeper shades of blue.

| **Biomechanical Metrics Included** | **PC1** | **PC2** | **PC3** | **PC4** | **PC5** | **PC6** | **PC7** | **PC8** |  |
| --- | --- | --- | --- | --- | --- | --- | --- | --- | --- |
| Total Impact Load | 0.64 | -0.27 | -0.40 | 0.56 | 0.05 | 0.13 | -0.03 | 0.06 |  |
| Total Step Count | 0.31 | -0.11 | -0.50 | 0.57 | -0.03 | 0.36 | -0.15 | -0.31 |  |
| Ave. Intensity | 0.68 | -0.32 | -0.16 | 0.35 | 0.11 | -0.13 | 0.04 | 0.31 |  |
| Impact Asym. | 0.46 | 0.79 | 0.15 | 0.24 | 0.04 | -0.07 | -0.02 | 0.08 |  |
| Low-G Asym. | 0.32 | 0.62 | -0.02 | 0.15 | -0.22 | -0.40 | -0.43 | -0.16 |  |
| Medium-G Asym. | 0.36 | 0.59 | 0.15 | 0.03 | 0.13 | 0.44 | 0.43 | 0.17 |  |
| High-G Asym. | -0.45 | -0.75 | -0.16 | -0.22 | -0.08 | 0.11 | -0.04 | -0.10 |  |
| Jump Height | 0.82 | -0.28 | 0.09 | -0.15 | 0.36 | -0.18 | -0.06 | -0.01 |  |
| CMD | -0.28 | 0.21 | -0.70 | -0.01 | -0.29 | -0.31 | 0.39 | -0.06 |  |
| Time to Takeoff | -0.26 | -0.32 | 0.71 | 0.47 | 0.17 | -0.06 | -0.08 | 0.02 |  |
| Pk Rel. Brk Power | -0.46 | -0.25 | 0.22 | 0.51 | -0.13 | -0.41 | 0.40 | -0.04 |  |
| Pk Rel. Prop Power | 0.82 | -0.20 | -0.05 | -0.11 | 0.34 | -0.27 | 0.14 | -0.03 |  |
| Pk Brk Force Asym. | -0.72 | 0.23 | -0.26 | 0.07 | 0.52 | -0.01 | -0.05 | 0.05 |  |
| Pk Prop Force Asym. | -0.75 | 0.11 | -0.18 | 0.07 | 0.48 | -0.10 | -0.07 | 0.06 |  |
| Ave. Brk RFD Asym. | -0.66 | 0.21 | -0.16 | 0.08 | 0.48 | -0.03 | 0.04 | -0.25 |  |
| Pk Lnd Force Asym. | -0.55 | -0.04 | -0.37 | 0.04 | -0.10 | -0.05 | -0.24 | 0.58 |  |
| RSI Mod. | 0.83 | -0.07 | -0.30 | -0.35 | 0.23 | -0.13 | 0.02 | -0.01 |  |
| *Individual % Var. Exp.* | *34* | *15* | *11* | *9* | *7* | *5* | *5* | *4* |  |
| *Cumulative % Var. Exp.* | *34* | *49* | *60* | *70* | *77* | *83* | *87* | *91* |  |
| Abbreviations: PC = principal component; Ave. = average; Asym. = asymmetry; CMD = countermovement depth; Pk = peak; Rel. = relative; Brk = braking; Prop = propulsive; RFD = rate of force development; Lnd = landing; RSI Mod. = the modified reactive strength index; % Var. Exp. = percent variance explained. | | | | | | | | | |


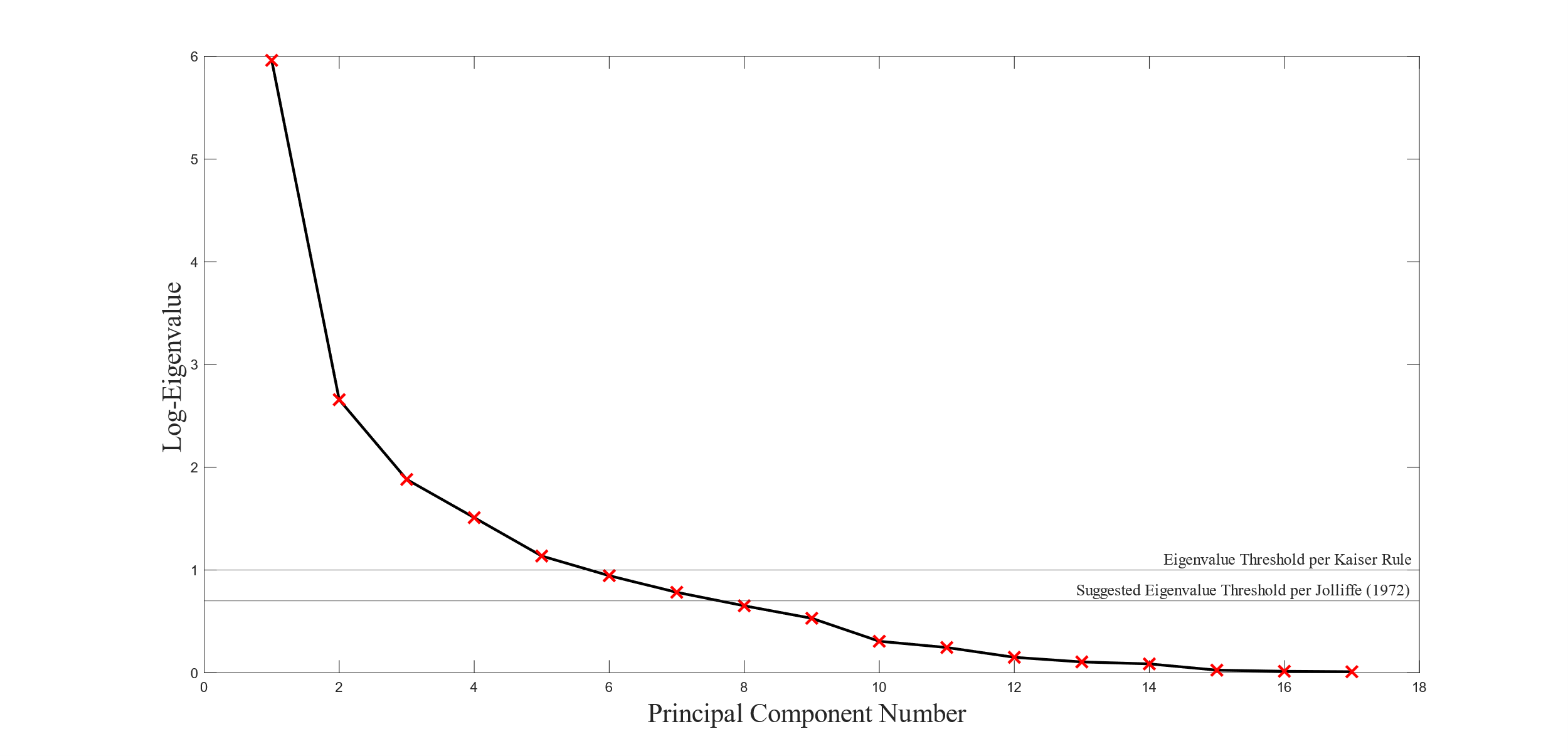


**Supplementary Figure 1.** Scree plot of log-eigenvalues of each principal component in the biomechanical PCA model vs. the number of principal components in the model.


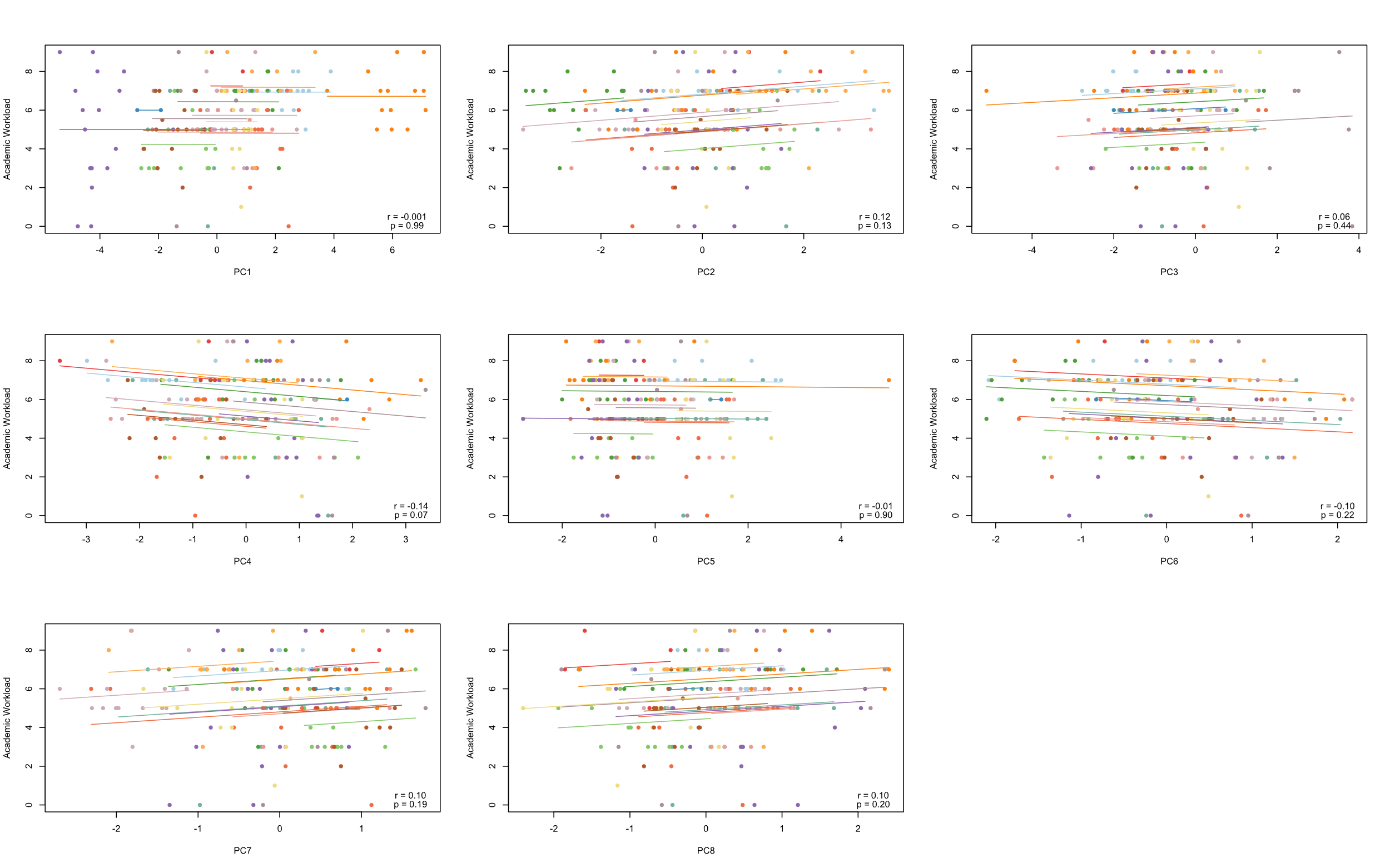


**Supplementary Figure 2.** Repeated measures correlation-based associations identified between biomechanical principal component scores and self-reported academic workload across the 2022-2023 season, with subject-specific data distinguished using different colours, and the commonality in within-individual associations after controlling for between-individual variance identified in the bottom right corner of each subplot.


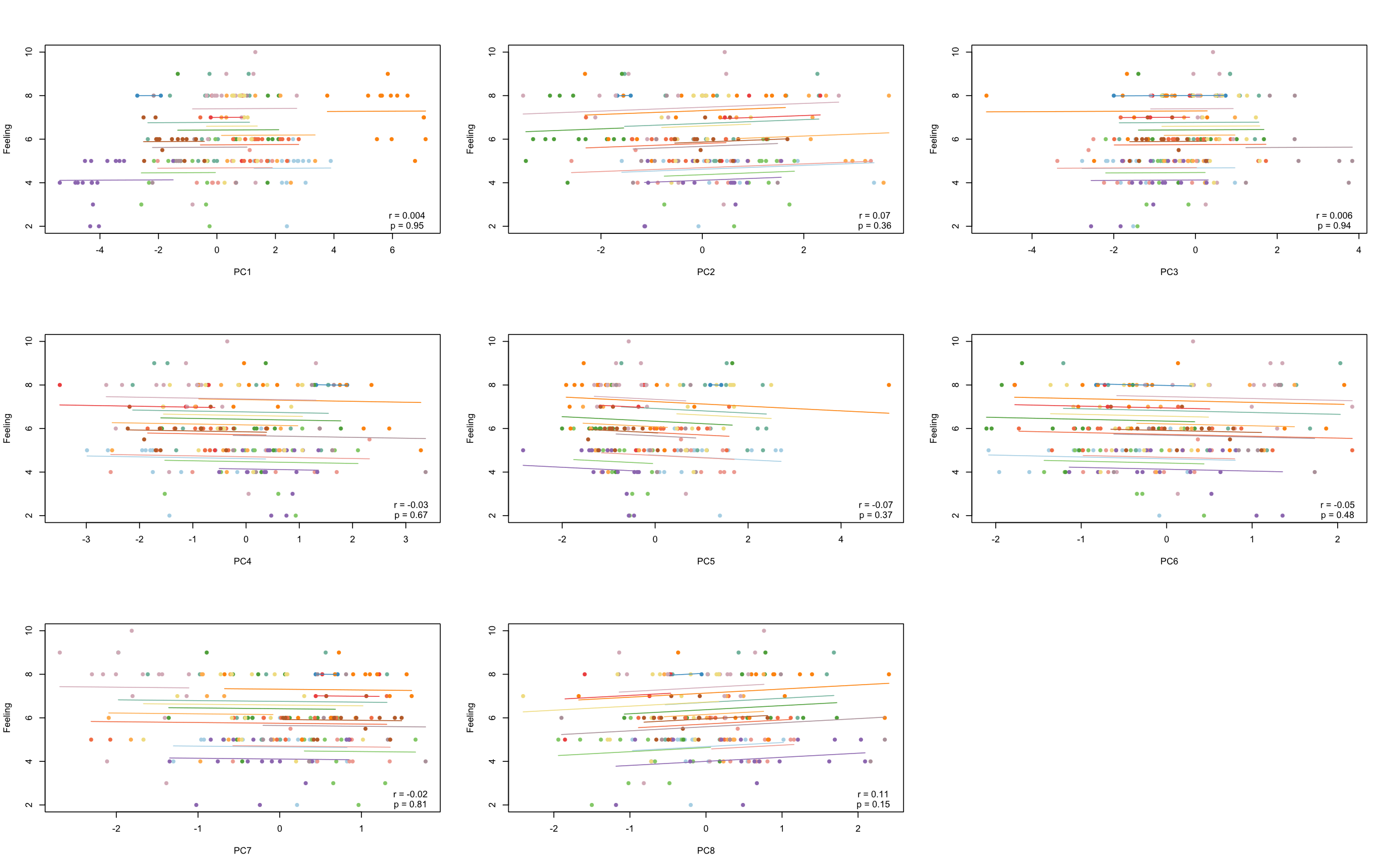


**Supplementary Figure 3.** Repeated measures correlation-based associations identified between biomechanical principal component scores and self-reported feeling across the 2022-2023 season, with subject-specific data distinguished using different colours, and the commonality in within-individual associations after controlling for between-individual variance identified in the bottom right corner of each subplot.


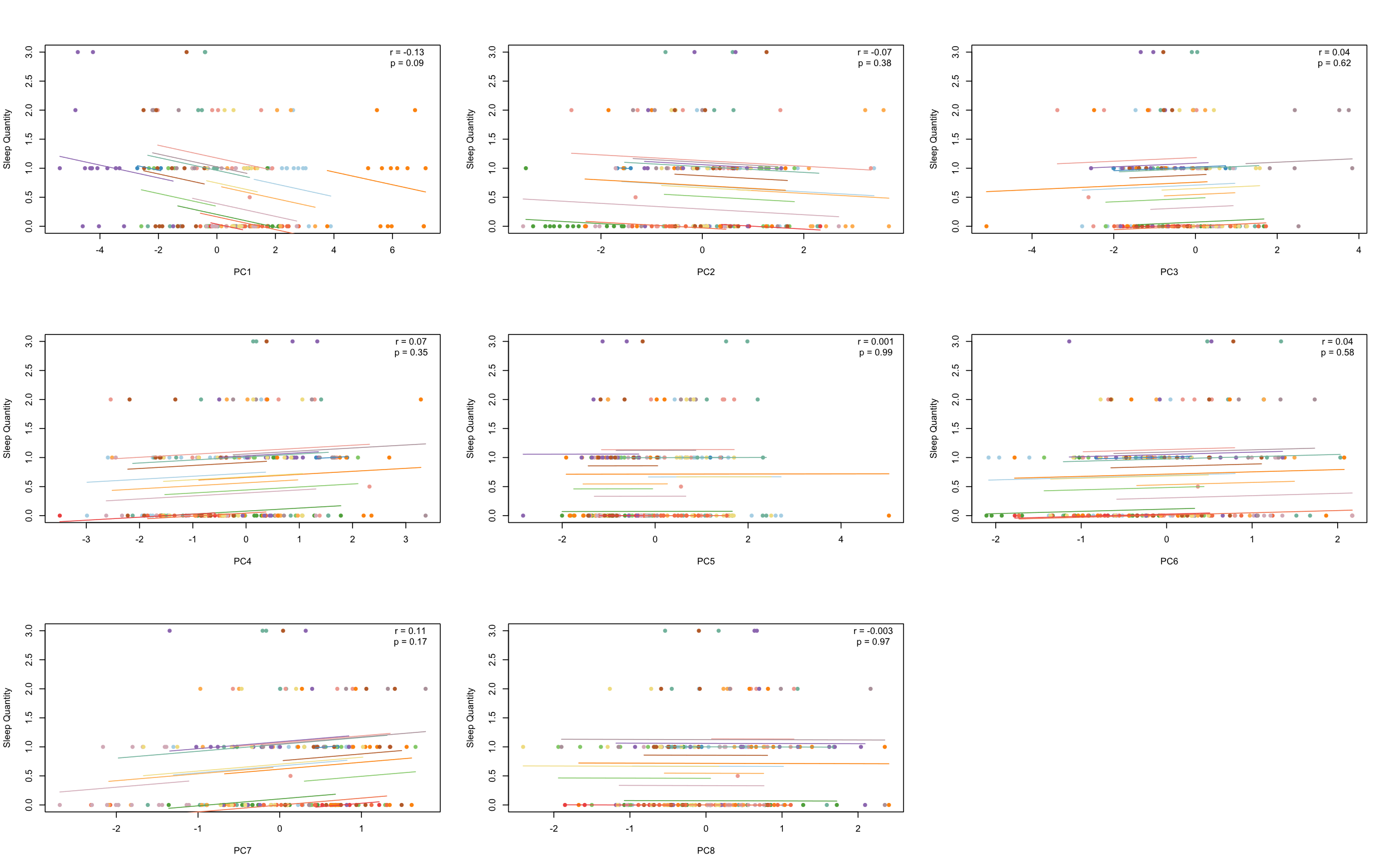


**Supplementary Figure 4.** Repeated measures correlation-based associations identified between biomechanical principal component scores and self-reported sleep quantity across the 2022-2023 season, with subject-specific data distinguished using different colours, and the commonality in within-individual associations after controlling for between-individual variance identified in the bottom right corner of each subplot.


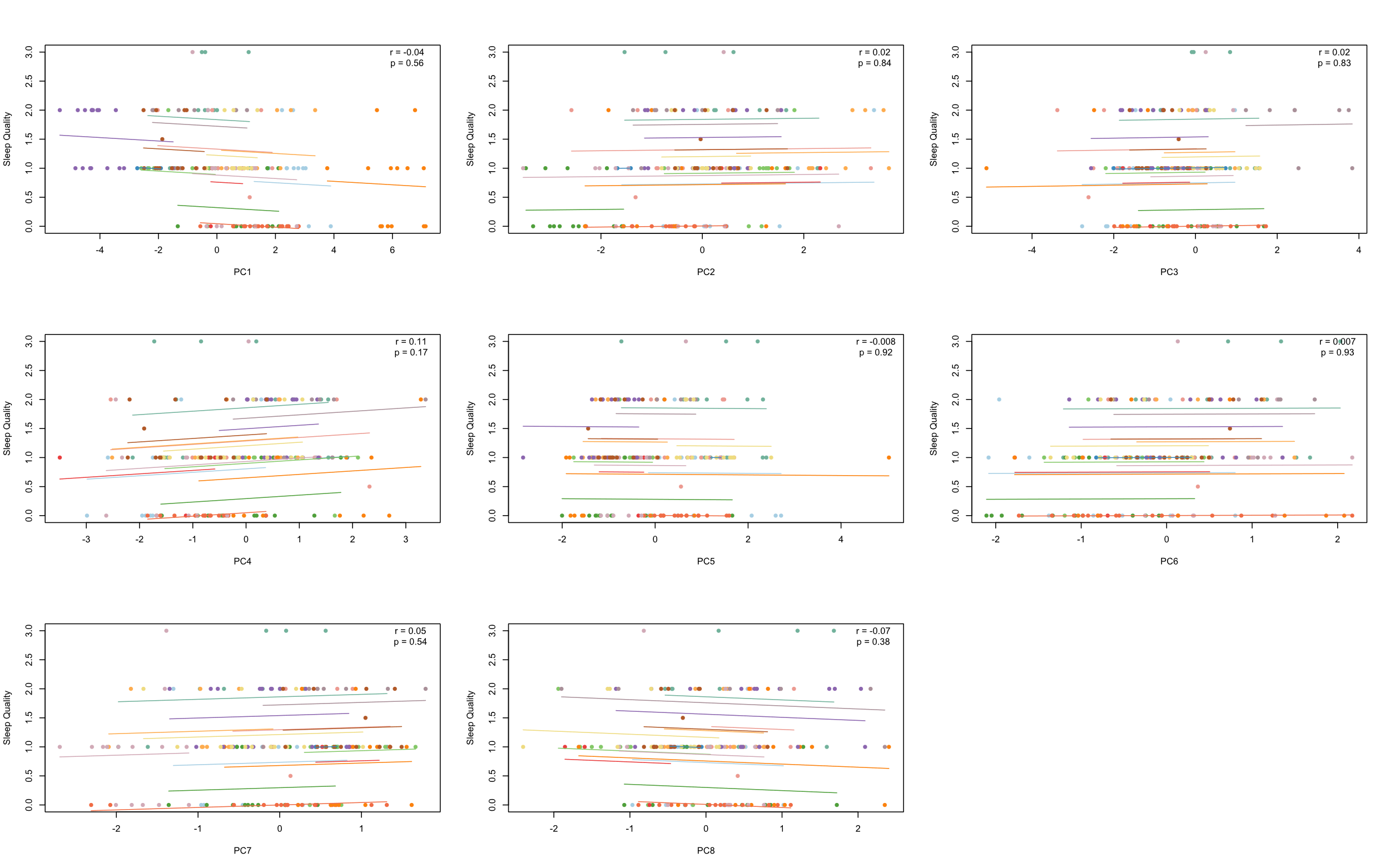


**Supplementary Figure 5.** Repeated measures correlation-based associations identified between biomechanical principal component scores and self-reported sleep quality across the 2022-2023 season, with subject-specific data distinguished using different colours, and the commonality in within-individual associations after controlling for between-individual variance identified in the bottom right corner of each subplot.


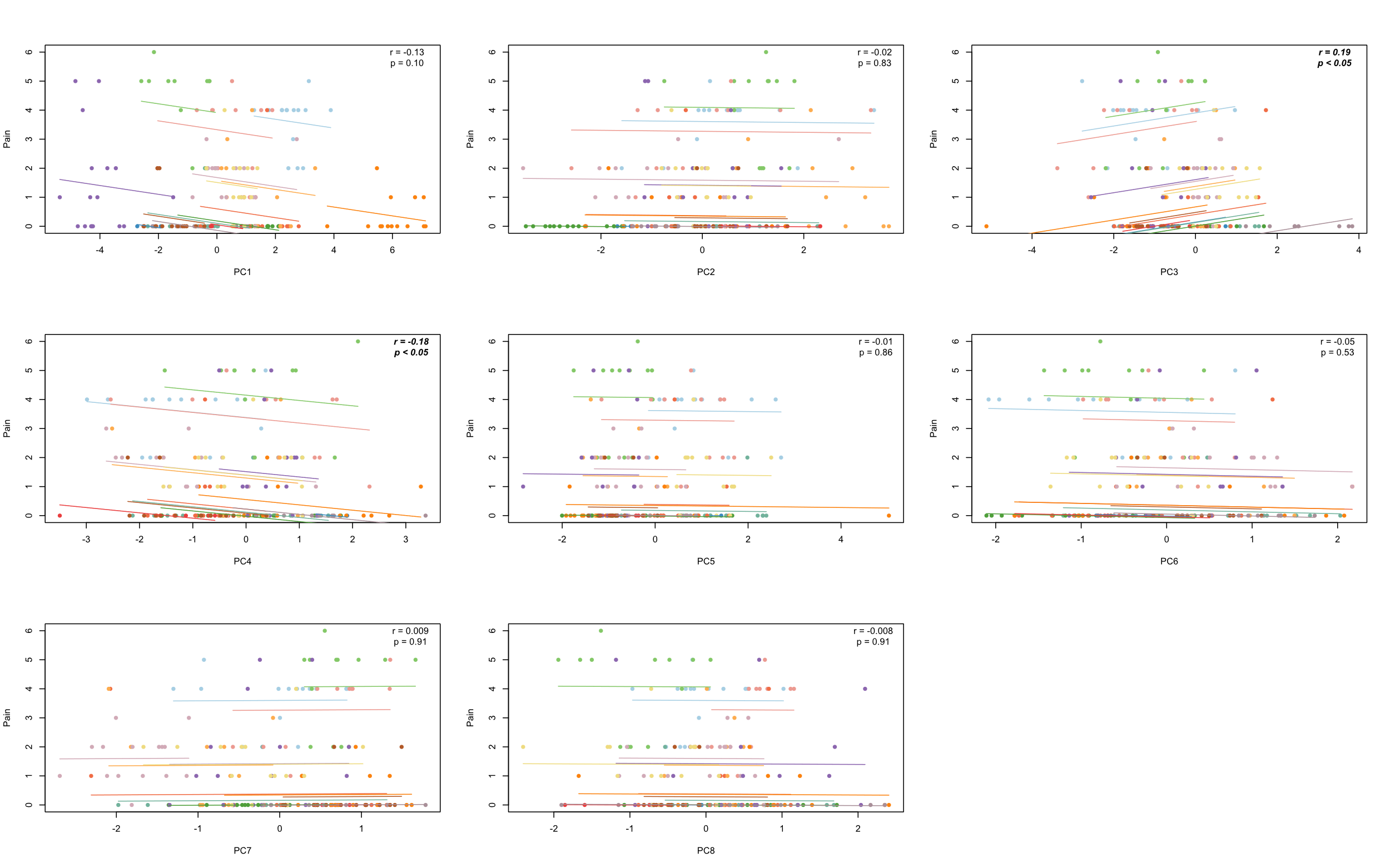


**Supplementary Figure 6.** Repeated measures correlation-based associations identified between biomechanical principal component scores and self-reported levels of pain across the 2022-2023 season, with subject-specific data distinguished using different colours, and the commonality in within-individual associations after controlling for between-individual variance identified in the bottom right corner of each subplot.


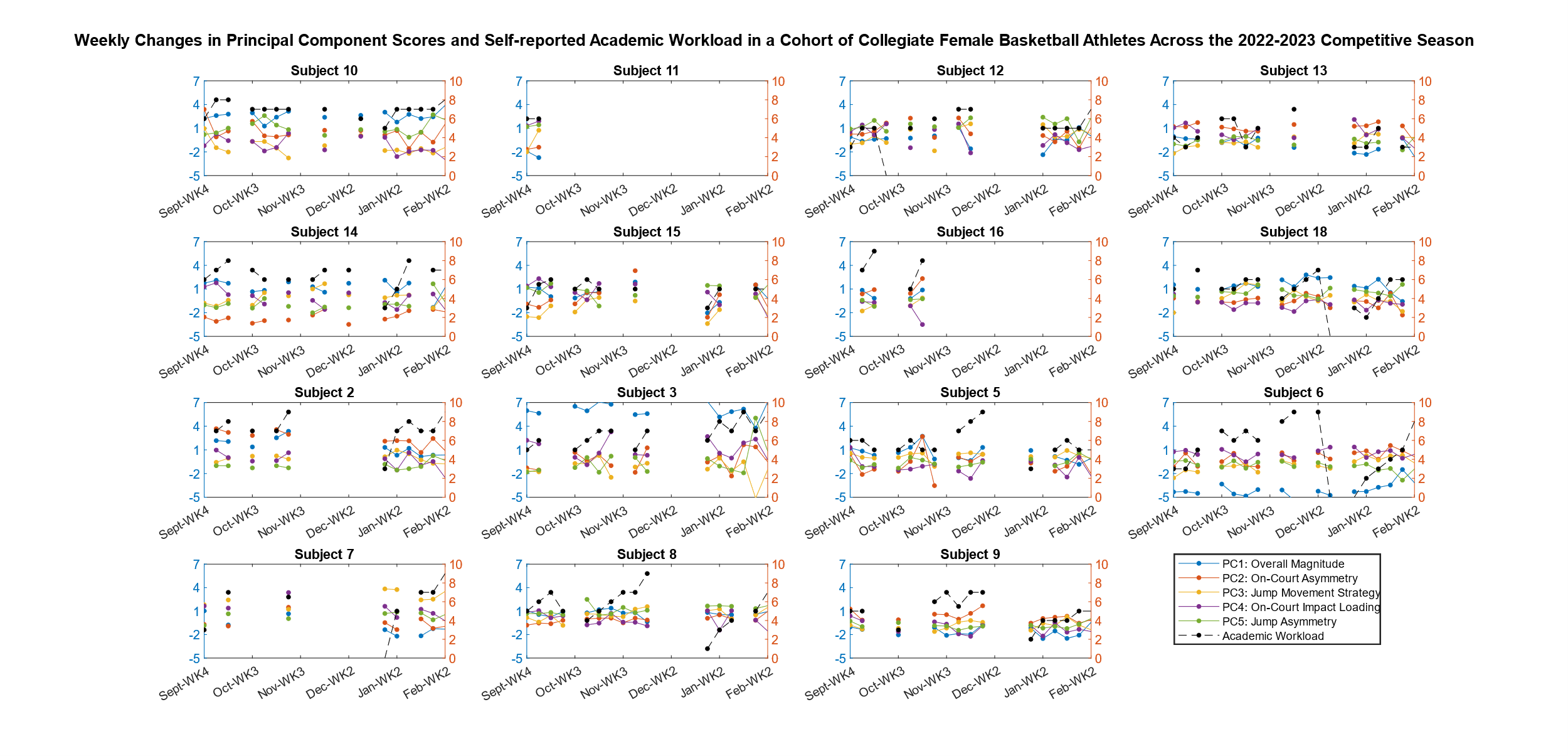


**Supplementary Figure 7.** Weekly changes in biomechanical principal component scores and self-reported academic workload in a cohort of collegiate female basketball athletes across the 2022-2023 competitive season.


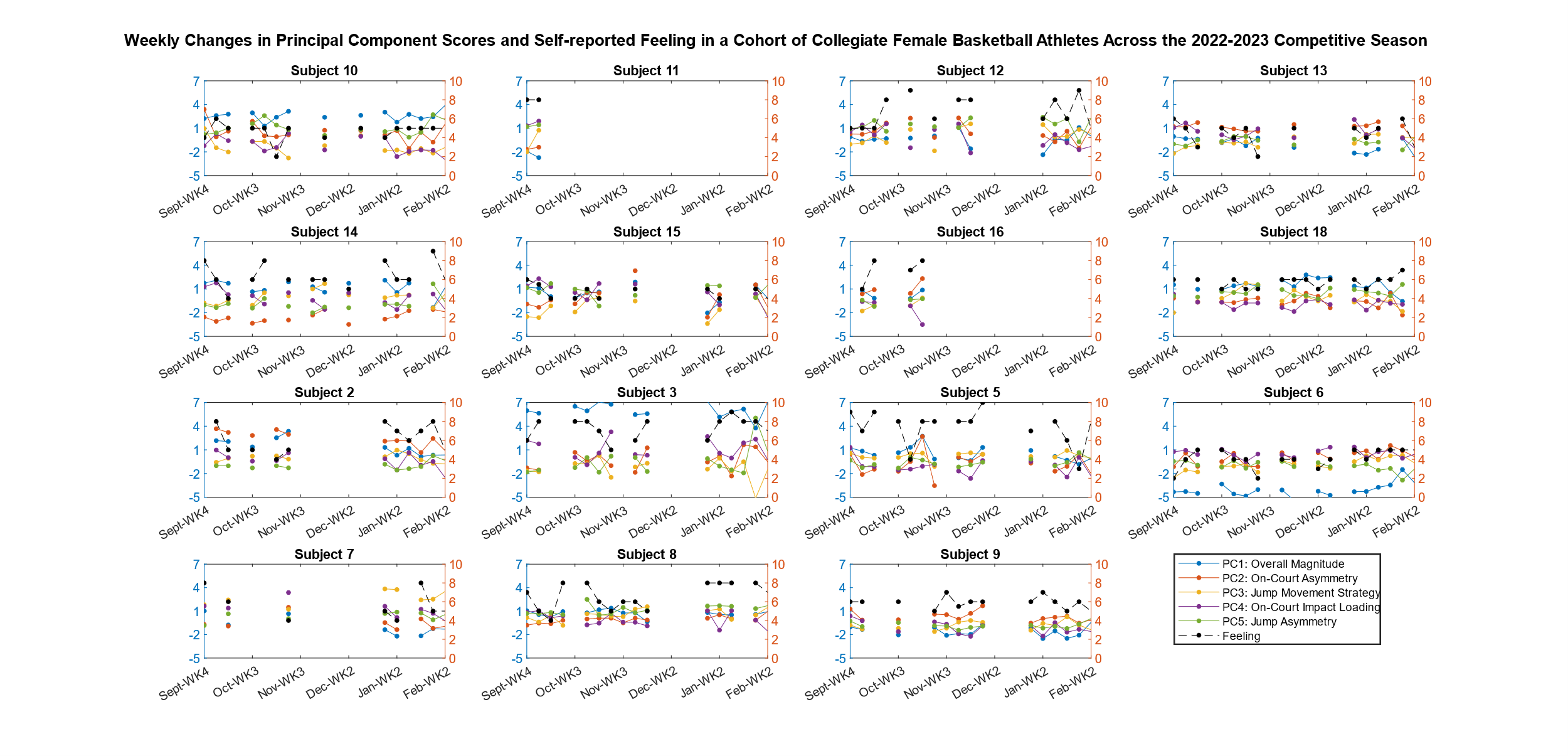


**Supplementary Figure 8.** Weekly changes in biomechanical principal component scores and self-reported feeling in a cohort of collegiate female basketball athletes across the 2022-2023 competitive season.


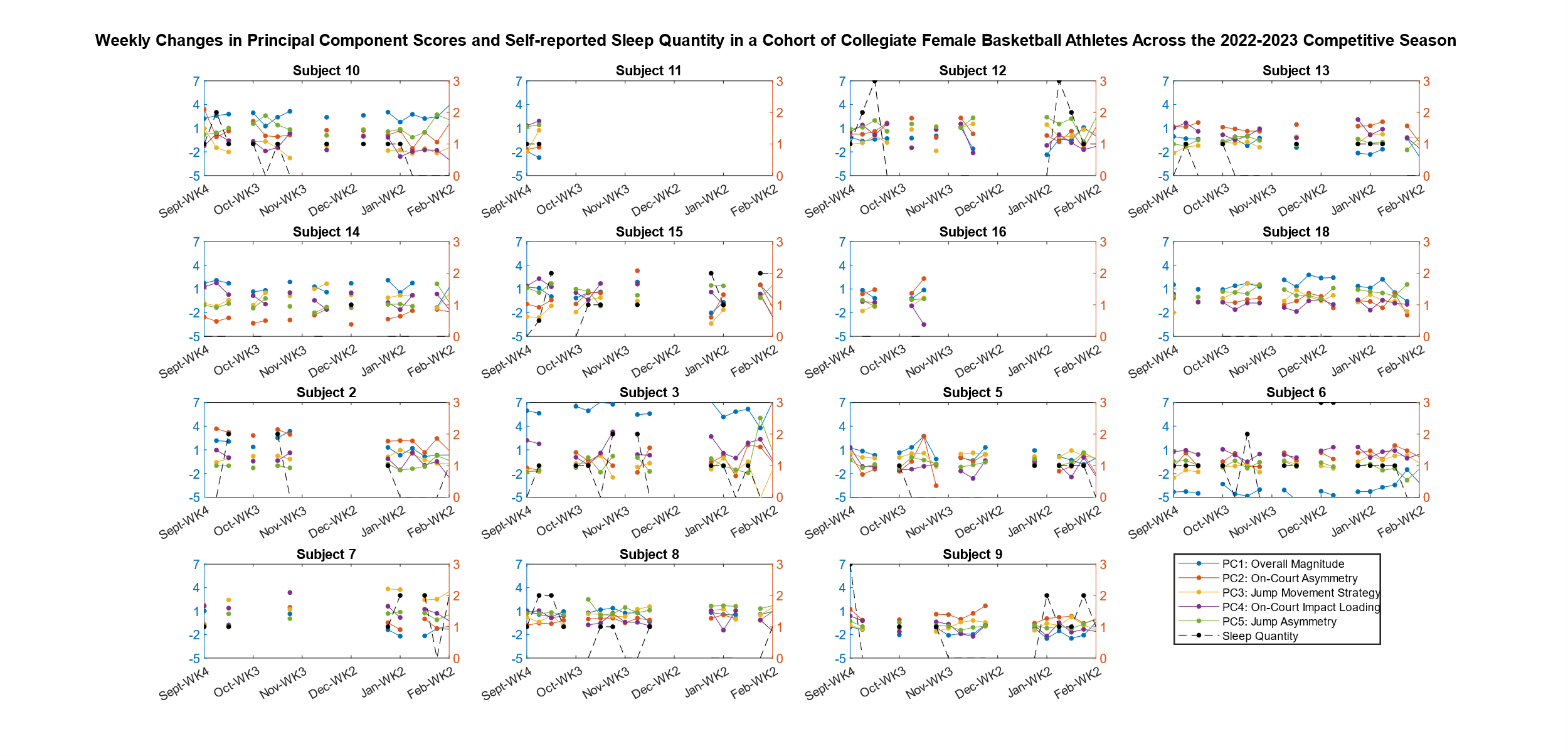


**Supplementary Figure 9.** Weekly changes in biomechanical principal component scores and self-reported sleep quantity in a cohort of collegiate female basketball athletes across the 2022-2023 competitive season.


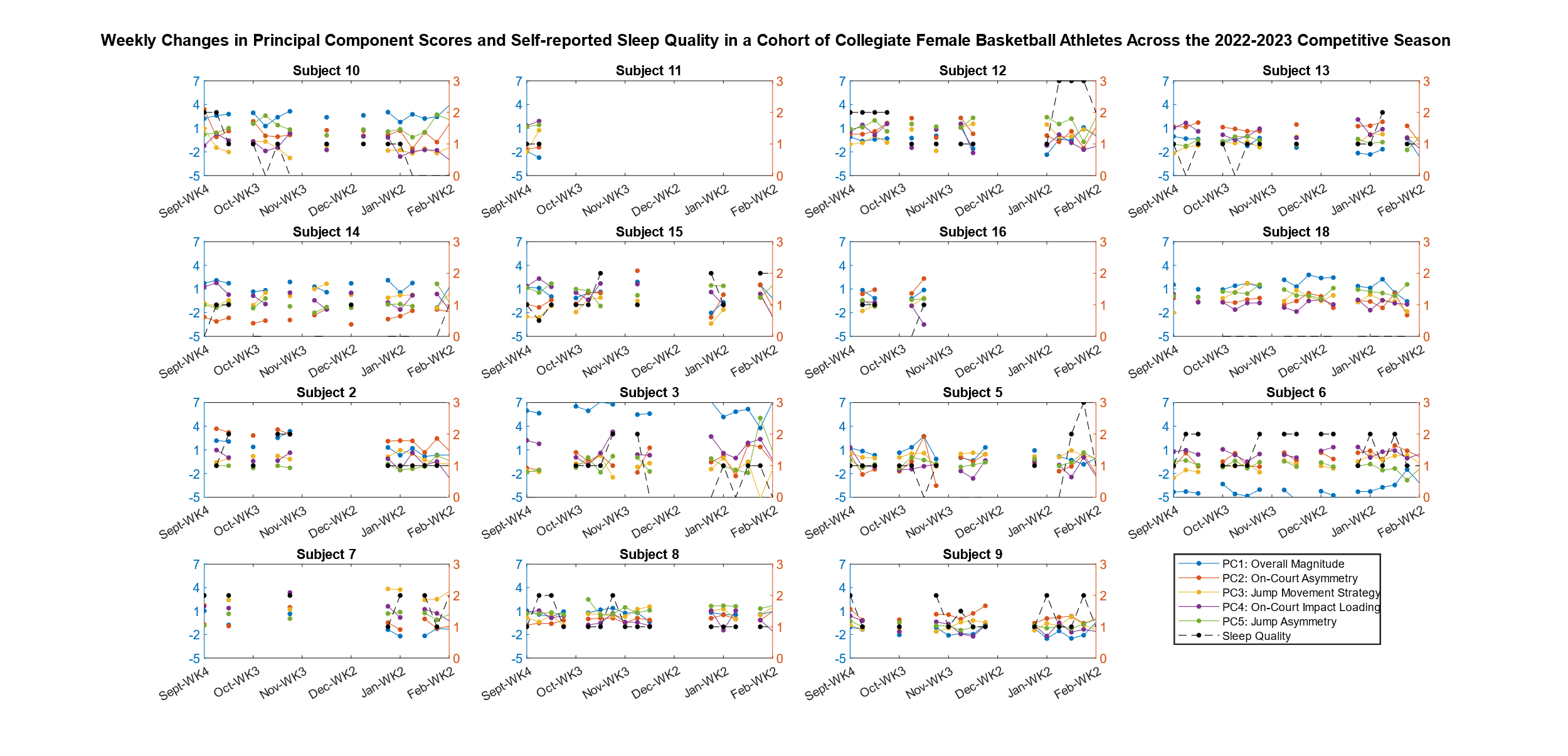


**Supplementary Figure 10.** Weekly changes in biomechanical principal component scores and self-reported sleep quality in a cohort of collegiate female basketball athletes across the 2022-2023 competitive season.


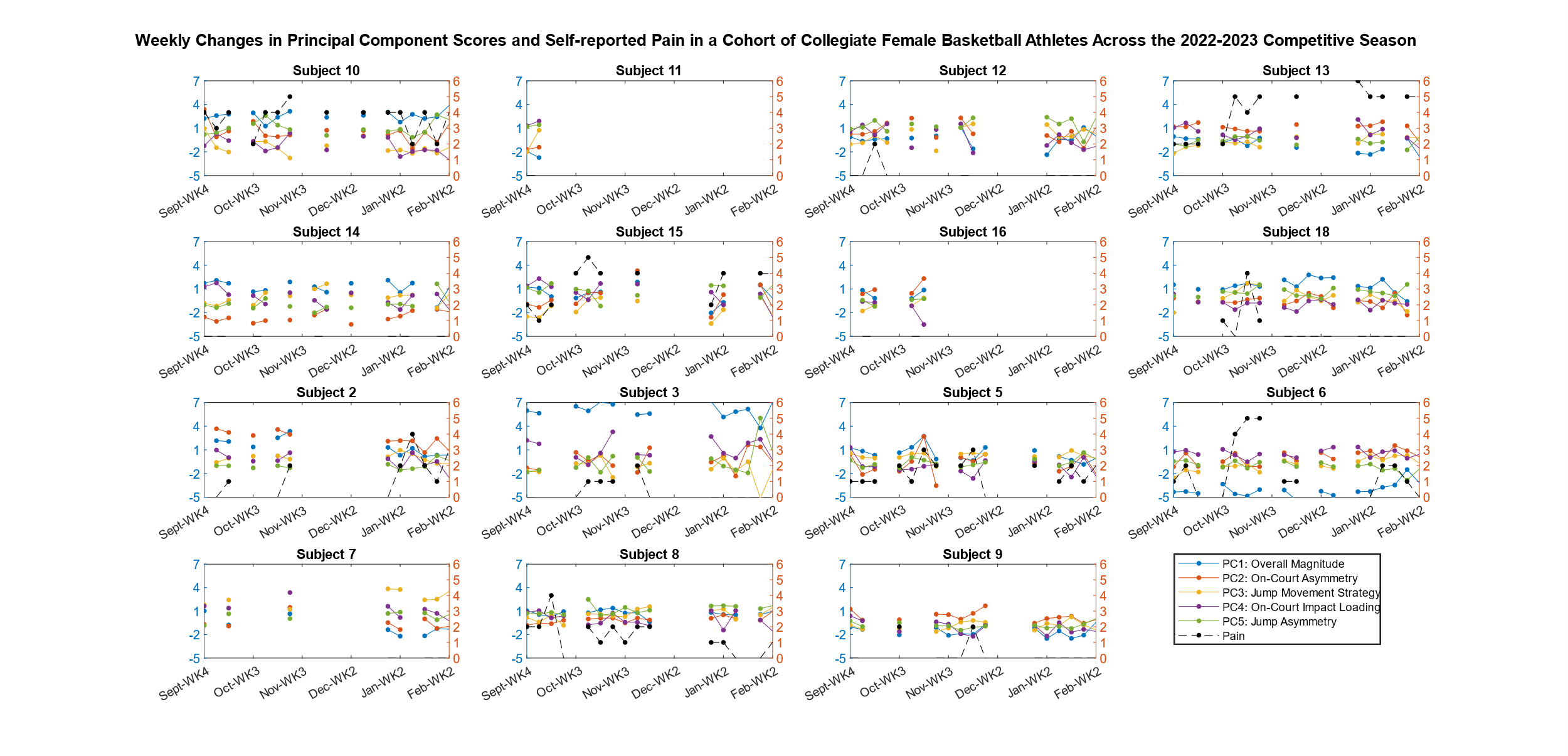


**Supplementary Figure 11.** Weekly changes in biomechanical principal component scores and self-reported pain in a cohort of collegiate female basketball athletes across the 2022-2023 competitive season.
